# Mechanisms Involved in the Functional Divergence of Duplicated GroEL Chaperonins in *Myxococcus xanthus* DK1622

**DOI:** 10.1371/journal.pgen.1003306

**Published:** 2013-02-21

**Authors:** Yan Wang, Wen-yan Zhang, Zheng Zhang, Jian Li, Zhi-feng Li, Zai-gao Tan, Tian-tian Zhang, Zhi-hong Wu, Hong Liu, Yue-zhong Li

**Affiliations:** State Key Laboratory of Microbial Technology, School of Life Science, Shandong University, Jinan, China; University of Michigan, United States of America

## Abstract

The gene encoding the GroEL chaperonin is duplicated in nearly 30% of bacterial genomes; and although duplicated *groEL* genes have been comprehensively determined to have distinct physiological functions in different species, the mechanisms involved have not been characterized to date. *Myxococcus xanthus* DK1622 has two copies of the *groEL* gene, each of which can be deleted without affecting cell viability; however, the deletion of either gene does result in distinct defects in the cellular heat-shock response, predation, and development. In this study, we show that, from the expression levels of different *groELs*, the distinct functions of *groEL1* and *groEL2* in predation and development are probably the result of the substrate selectivity of the paralogous GroEL chaperonins, whereas the lethal effect of heat shock due to the deletion of *groEL1* is caused by a decrease in the total *groEL* expression level. Following a bioinformatics analysis of the composition characteristics of GroELs from different bacteria, we performed region-swapping assays in *M. xanthus*, demonstrating that the differences in the apical and the C-terminal equatorial regions determine the substrate specificity of the two GroELs. Site-directed mutagenesis experiments indicated that the GGM repeat sequence at the C-terminus of GroEL1 plays an important role in functional divergence. Divergent functions of duplicated GroELs, which have similar patterns of variation in different bacterial species, have thus evolved mainly via alteration of the apical and the C-terminal equatorial regions. We identified the specific substrates of strain DK1622's GroEL1 and GroEL2 using immunoprecipitation and mass spectrometry techniques. Although 68 proteins bound to both GroEL1 and GroEL2, 83 and 46 proteins bound exclusively to GroEL1 or GroEL2, respectively. The GroEL-specific substrates exhibited distinct molecular sizes and secondary structures, providing an encouraging indication for GroEL evolution for functional divergence.

## Author Summary

GroEL is a type I chaperonin, involved in protein folding, assembly, and transport. It is a major group of heat-shock proteins that are over-expressed at high temperatures and has fundamental roles in growth and survival at non-permissive temperatures. Because of its importance in many cellular processes, the *groEL* gene is ubiquitously distributed in bacteria. Most bacterial species possess a single *groEL* gene, while others (close to 30% of sequenced bacterial genomes) have two or more *groEL* copies. Many studies have described the functional divergence of duplicated *groEL* genes in different bacterial species, but the involved mechanisms have not yet been characterized. Myxobacteria are characterized by their unique multicellular behaviors. *Myxococcus xanthus* DK1622, the model strain of myxobacteria, possesses a large genome (9.14 Mb), containing many gene duplications, including two copies of the *groEL* gene. Gene duplications and their functional divergence are suggested for complex cellular behaviors, which, however, have not yet been testified. In this paper, using combined proteomic and genetic approaches, we explored how the duplicated *groEL* genes of *M. xanthus* DK1622 evolved to fit the functional divergence for social behaviors.

## Introduction

Chaperonins are essential cellular components that are responsible for protein folding, assembly and transport [1]–[6]. Chaperonins are also a major group of heat shock proteins that are over-expressed at high temperatures and have fundamental roles in growth and survival at non-permissive temperatures [6]–[8]. GroEL is a type I chaperonin, and in *Escherichia coli*, the GroEL chaperonin is required *in vivo* for the proper folding, at all temperatures, of approximately 300 newly translated polypeptides (accounting for approximately 10% of the total) that participate in various physiological processes [9]. Because of its importance in many cellular processes, the *groEL* gene is ubiquitously distributed in bacteria. Most bacterial species, such as *E. coli*, possess a single *groEL* gene, whereas other species (nearly 30% of bacteria with sequenced genomes) have evolved multiple *groEL* copies [1]. The paralogous GroEL proteins are highly similar in sequence and, most likely, in structure. However, some differences exist between duplicated *groEL* genes, and these duplicated GroEL proteins have evolved to play divergent roles in many different cellular processes in different bacterial species [10]–[15]. Although the mechanisms of functional divergence are important for our understanding of the complexity of evolution, these mechanisms have not been characterized to date.

Myxobacteria are δ-proteobacteria with unique and complex multicellular behaviors, such as movement in swarms on solid surfaces, cooperative feeding on macromolecules or other microbial cells and the development of multicellular fruiting bodies containing numerous myxospores against adversity conditions [16], [17]. *Myxococcus xanthus* DK1622 is a model myxobacterium with a large genome (9.14 Mbp) that includes many duplicated crucial genes [18]. It has been suggested that such duplication is responsible for the complex social behavior of these cells, although this hypothesis has not been experimentally validated. There are two copies of the *groEL* gene in the genome of *M. xanthus* DK1622. Previous studies indicate that either of the two paralogous *groEL* genes can be deleted in strain DK1622 without affecting cellular viability, although the deletion does result in distinct defects in the cellular heat-shock response, predation and development [15]. In this study, we investigated the effects of the substrate selectivity of the GroEL proteins and the expression levels of the duplicated *groEL* genes on the functional divergence of heat-shock responses, development and predation. We performed a comparative proteomics analysis of the substrate specificity of the two GroELs, and the relationships between the structural differences and substrate specificity were investigated using bioinformatics, molecular swapping and site-directed mutagenesis.

## Results

### Effects of *groEL* expression levels on functional divergence

**Figure 1:**
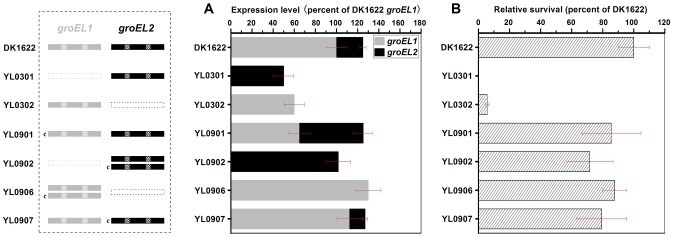
Quantitative PCR analysis of the *groEL1* and *groEL2* expression levels and survival rates after heat shock at 42°C for 30 min. (A) Gene expression levels. (B) Survival rates after heat shock. The left panel is a schematic diagram of the *groELs* gene present in the different strains. The values for each *groEL* gene are shown as levels relative to the expression of *groEL1* in DK1622, which was defined as 100%. The survival rates of the different strains were calculated as a percentage of the survival rate of DK1622, which was 1.01×10^−2^ after the heat shock treatment and was defined as 100%. The error bars show the standard deviation of three replicates. DK1622, wild-type strain; YL0301, *groEL1*-deletion mutant; YL0302, *groEL2*-deletion mutant; YL0901, YL0301 complemented with GroEL1; YL0902, YL0301 complemented with GroEL2; YL0906, YL0302 complemented with GroEL1; YL0907, YL0302 complemented with GroEL2. “c” denotes “complement.”

Otani *et al.* found that, although GroEL1 and GroEL2 are among the major proteins induced by heat shock, the density of GroEL2 spots in two-dimensional electrophoretic gels is much lower than that of GroEL1 [19]. It was also noted that the expression levels of *groEL1* and *groEL2* were not equal in the wild-type strain DK1622 in CTT growth medium and TPM development medium and that the two *groEL* genes played distinct roles in heat-shock responses, development and predation [15]. To assess the changes in *groEL1* or *groEL2* expression in *groEL*-deletion mutants and whether these changes contribute to functional divergence, we inserted *groEL1* or *groEL2*, each with its own promoter, into the genome of *groEL1*- or *groEL2*-deletion mutants at the *attB* integration site using pSWU30, producing four *groEL*-complemented strains (Table S1). The *groEL* expression levels and cell viability were compared between these mutants and the wild-type strain DK1622 following heat shock at 42°C for 30 min. Quantitative PCR assays indicated that the expression of the *groEL* genes was regulated in a complex manner in different mutants upon heat shock (Figure 1). In the wild-type strain DK1622, the *groEL2* expression level was only one-quarter of that of *groEL1* after heat shock. The deletion of *groEL1* (strain YL0301) led to an increase in the *groEL2* expression level (approximately twofold). The expression of *groEL1* inserted in YL0301 (strain YL0901) was approximately half that in DK1622 under the heat shock conditions, but the presence of exogenous *groEL1* had no obvious effect on the expression level of *groEL2* (*P*>0.05). Thus, the total expression of all the *groEL* genes in YL0901 was similar to that in DK1622. In YL0902, which contained an additional *groEL2* gene, the total expression of *groEL2* also doubled, reaching a level equal to four times that of *groEL2* in DK1622. In YL0302, the deletion of the *groEL2* gene led to reduced expression of the *groEL1* gene under the heat shock conditions (approximately 60% of that in DK1622). Transformation of the YL0302 mutant (strain YL0906) with another *groEL1* gene increased the total *groEL* expression level to that of DK1622 (*P*>0.05). The total expression level of *groEL1* and *groEL2* in the *groEL2*-complemented YL0302 mutant (strain YL0907) also reached the level in DK1622 (*P*>0.05).

Interestingly, although YL0902 had two *groEL2* genes and no *groEL1*, the survival rates of both YL0901 and YL0902 were similarly increased after heat shock at 42°C for 30 min, paralleling the increase in the total *groEL* expression level in these two mutants (Figure 1). The survival rates of the YL0906 and YL0907 mutants after the heat shock treatment also corresponded to an increase in the total *groEL* expression level. These results suggest that the lethal nature of the heat shock in the *groEL1* deletion mutant (YL0301) and the increased sensitivity of the *groEL2* deletion mutant (YL0302) to high temperatures [15] result from a significant decrease in the total expression of GroEL, leading to an insufficient level of GroEL proteins to facilitate the refolding of denatured proteins. This is consistent with a model where there is a threshold level of GroEL beneath which cells cannot survive. When the total expression of *groEL* is higher than the threshold, there is a positive correlation between the survival rate and *groEL* expression in *M. xanthus* DK1622 cells after heat shock (r = 0.98, *P*<0.01) (Figure S1), a result which supports the hypothesis that, after duplication, both *groEL1* and *groEL2* retain fundamental functions by balancing their expression dosage [20], [21].

**Figure 2:**
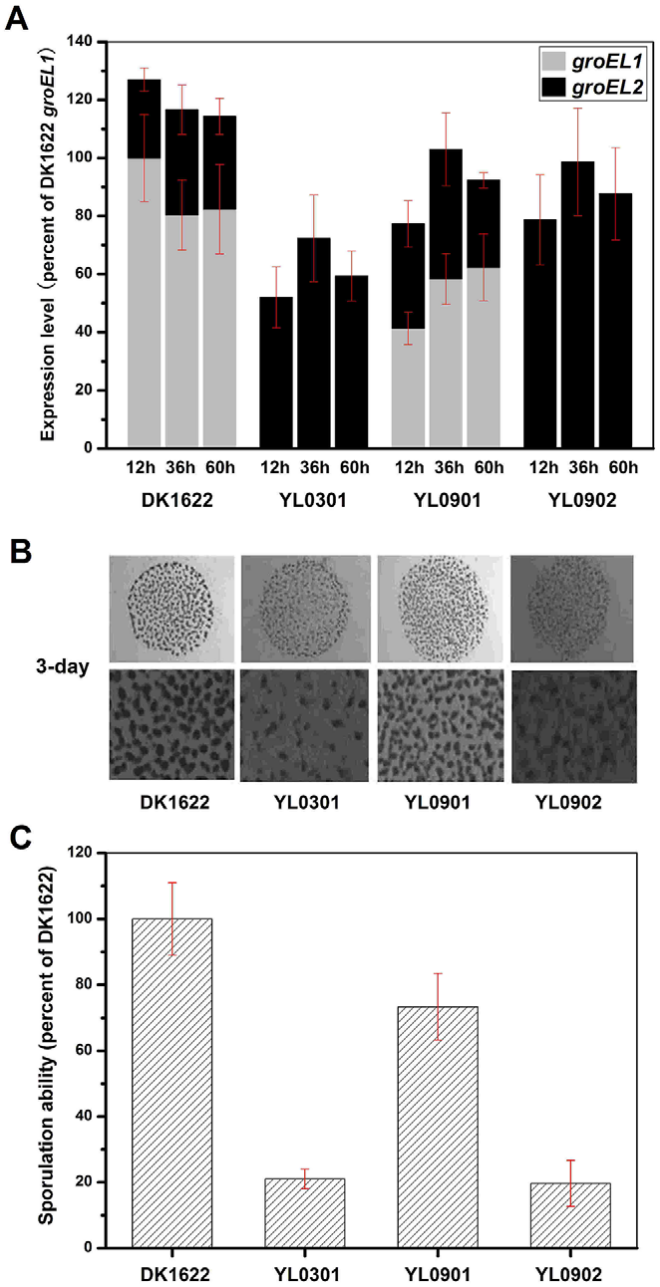
The expression levels of *groEL* genes, the development and sporulation analysis of different strains. (A) The expression levels of *groEL* genes at three developmental time points. (B) The development of fruiting bodies. (C) The sporulation of different strains on TPM plates after three days. The expression levels for each *groEL* gene in the different strains are shown as levels relative to the expression of DK1622 *groEL1* after 12 hours, which was defined as 100%. The sporulation ability of the different strains was calculated as a percentage of the sporulation by DK1622, which was defined as 100%. DK1622, wild-type strain; YL0301, *groEL1*-deletion mutant; YL0901, YL0301 complemented with GroEL1; YL0902, YL0301 complemented with GroEL2; YL0903, YL0301 complemented with GroEL2-equatorial-N_GroEL1_; YL0904, YL0301 complemented with GroEL2-apical_GroEL1_; YL0905, YL0301 complemented with GroEL2-equatorial-C_GroEL1_.

Because the deletion of *groEL1* and the deletion of *groEL2* result in deficiencies in development and predation, respectively [15], we performed a development assay on DK1622, YL0301, YL0901 and YL0902 and predation assays on DK1622, YL0302, YL0906 and YL0907. Figure 2A shows the expression levels of *groEL1* and *groEL2* in different strains after 12, 36 and 60 h of incubation on TPM development medium. When *groEL1* was inserted into the genome of the *groEL1*-deletion mutant (strain YL0901), the developmental defect was mostly reversed, with sporulation reaching 70%–80% of that of DK1622. However, although YL0902 (containing two copies of *groEL2*) had a total *groEL* expression level similar to that of YL0901 at different developmental stages, YL0902 displayed a development defect similar to that of YL0301, and the sporulation ability of YL0902 was only approximately 20% of that of the wild-type strain DK1622 (Figure 2B and 2C).

**Figure 3:**
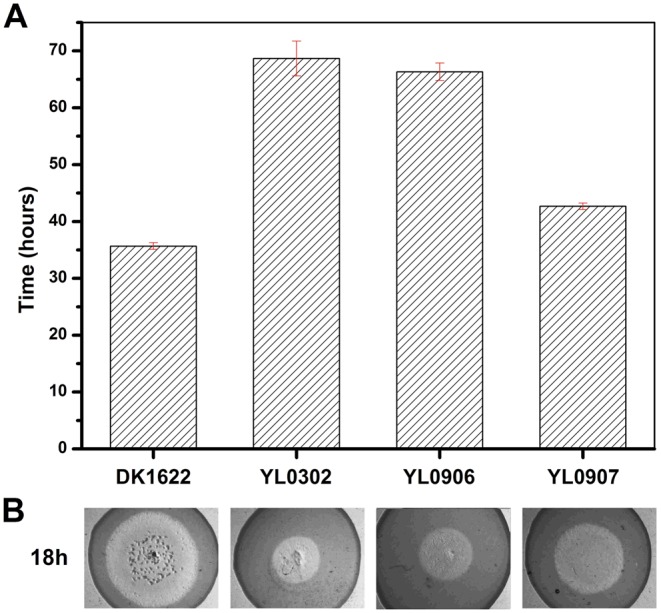
The predation of an *E. coli* mat by different strains. (A) The time for the different strains to reach the edge of the *E. coli* mat. (B) Zones of predation after 18 h and 36 h for the knockout and complemented mutants. DK1622, wild-type strain; YL0302, *groEL2*-deletion mutant; YL0906, YL0302 complemented with GroEL1; YL0907, YL0302 complemented with GroEL2; YL0908, YL0302 complemented with GroEL1-equatorial-N_GroEL2_; YL0909, YL0302 complemented with GroEL1-apical_GroEL2_; YL0910, YL0302 complemented with GroEL1-equatorial-C_GroEL2_.

The insertion of *groEL1* into YL0302 (strain YL0906) did not improve the predation feeding ability of cells on an *E. coli* mat, and the swarming time of YL0906 to the edge of the *E. coli* colony was 60–65 h, which is similar to that of YL0302 (*P*>0.05). When *groEL2* was inserted into YL0302 (strain YL0907), the swarming time to the *E. coli* colony edge decreased to 40 h (Figure 3). Because the presence of living *E. coli* cells in the *E. coli* predation experiments might affect the qPCR assay, we instead performed the analysis using a liquid feeding assay with casein as the only nutrient [15]. The total expression level of *groEL1* and *groEL2* was also similar in the YL0906 and YL0907 mutants, suggesting that the changes in expression are not the major contributors to functional divergence (Figure S2).

### Evolutionary analysis of duplicated GroELs

**Figure 4:**
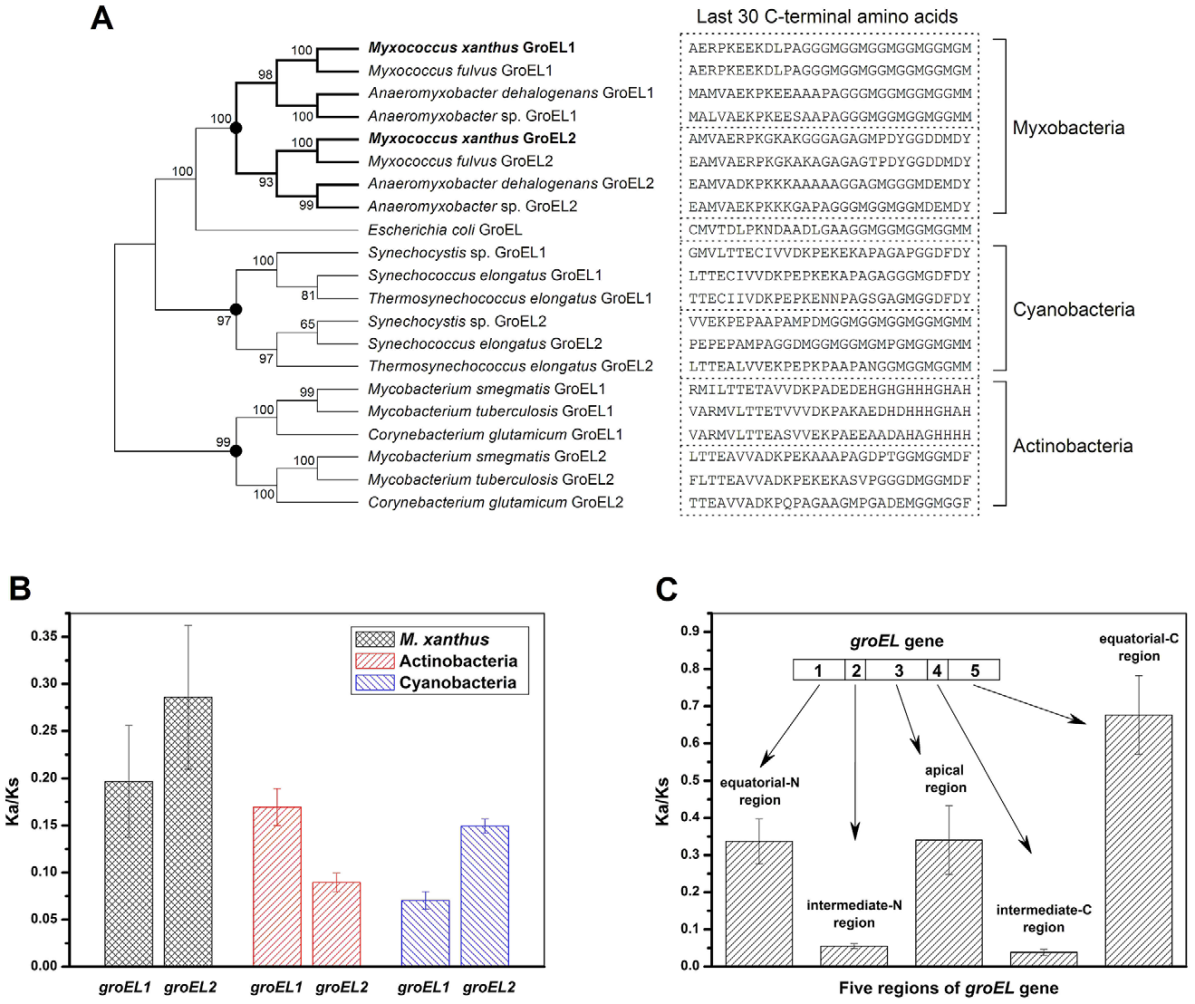
Evolutionary analysis of GroELs. (A) Maximum likelihood tree of the *M. xanthus* GroELs and the GroELs from ten other bacteria. The sequence of the last 30 C-terminal amino acids of each GroEL is shown. The black dots in the tree represent potential duplication events. (B) Mean Ka/Ks values of the different bacteria, as calculated using orthologous *groEL* genes. (C) Mean Ka/Ks values of five regions of the duplicated *groEL* genes from four myxobacterial species, as calculated using paralogous *groEL* genes.

The above results indicate that although the distinct heat-shock responses of the *groEL1* and *groEL2* mutants were determined by the total *groEL* expression level, the divergent functions of *groEL1* and *groEL2* in development and predation are the result of the substrate specificity of the corresponding GroEL chaperonins. To explore the evolutionary relationships of *groEL*, we compared the *M. xanthus* GroEL sequences with those of ten genome-sequenced bacteria, including three Myxobacteria, three Actinobacteria, three Cyanobacteria, and *E. coli* (Figure 4 and Table S2). With the exception of *E. coli*, these species possess duplicated *groEL* genes. The maximum likelihood tree showed that the GroELs from Myxobacteria, Actinobacteria and Cyanobacteria clustered separately (Figure 4A), suggesting that the *groEL* gene duplication originated from three independent evolutionary events in these three taxa. We further calculated the Ka/Ks values of these orthologous *groEL* genes (Figure 4B and Table S3). The average Ka/Ks values for Actinobacteria *groEL2* and Cyanobacteria *groEL1* were less than 0.1, suggesting that they are highly evolutionarily conserved. It has been reported that *groEL2* in the three Actinobacteria species [12], [22]–[24] and *groEL1* in the three Cyanobacteria species [25]–[27] are housekeeping genes, which is consistent with their Ka/Ks values. In *M. xanthus*, the Ka/Ks value for *groEL1* was significantly lower than that for *groEL2* (*P*<0.05) but significantly higher than that for the housekeeping *groEL* genes in Actinobacteria or Cyanobacteria (*P*<0.01). These results suggest that both of *groEL1* and *groEL2* in *M. xanthus* are suffered weak selection pressure, consistent to the finding that the deletion of either gene alone does not affect cell viability [15].

Based on their structural characteristics and sequence conservation, the GroEL protein sequences have been divided into five regions, *i.e.*, an N-terminal equatorial region, an N-intermediate region, an apical region, a C-intermediate region and a C-terminal equatorial region (Figure S3) [28]. The two intermediate regions have the highest level of conservation between *M. xanthus* DK1622 GroEL1 and GroEL2, *i.e.*, 97.7% and 97.2% identities for the N- and C-intermediate regions, respectively. The identities for the other three regions are 81% for the N-terminal equatorial region, 75.4% for the apical region, and 62.6% for the C-terminal equatorial region. Further Ka/Ks analysis showed similar sequence characteristics for the five GroEL1 and GroEL2 regions in the four Myxobacterial species referred to above (Figure 4C). For example, the Ka/Ks values of the N- and C-intermediate regions were very low (<0.05), suggesting that these two regions are highly conserved; in contrast, the other three regions had higher Ka/Ks values (>0.3), suggesting these regions are most likely involved in the functional divergence of GroEL1 and GroEL2. A sequence alignment showed that the high Ka/Ks values of the C-terminal equatorial regions were largely due to the variability of the C-terminal tail sequences. For example, the C-terminal tail of GroEL1 in *M. xanthus* was composed of six repeated GGM motifs, similar to that of *E. coli* GroEL, whereas the C-terminal tail of GroEL2 is greatly different. It was also noted that there are substantial differences in the C-terminal sequences between the duplicated GroELs (Figure 4A) [1].

### Region swapping and site-directed mutagenesis assays

**Figure 5:**
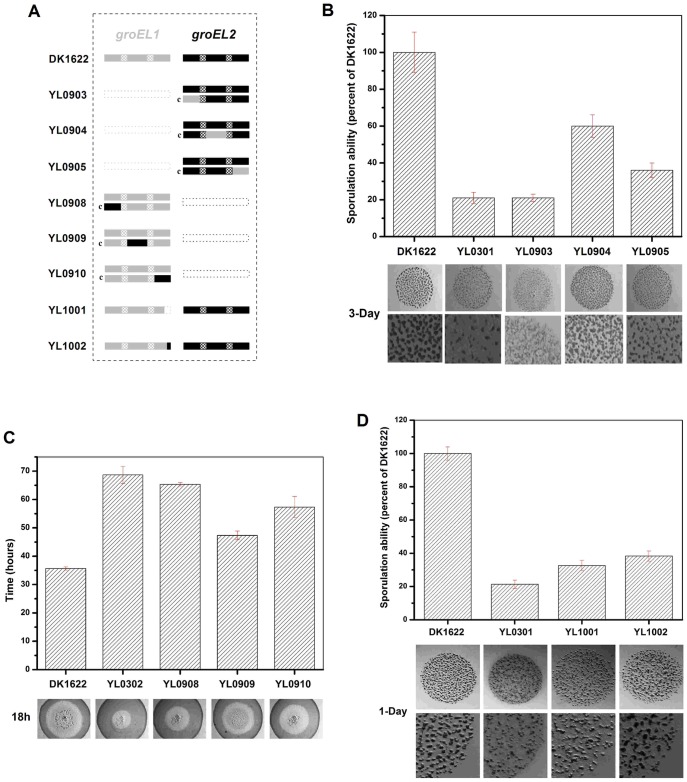
Social behavior feature of region-swapping mutants, GGM deletion, and shorten mutants. (A) is a schematic diagram of the *groELs* present in the different strains. (B) Sporulation ability and development of region-swapping mutants. (C) Predation speed of region-swapping mutants. (D) Sporulation ability and development of GGM deletion and shorten mutants. The sporulation ability of each strain was calculated as a percentage of the sporulation ability of DK1622, which was defined as 100%.

To clarify the relationships between the structural and functional divergence, we designed a series of single region-swaps between the *groEL1* and *groEL2* genes to determine the roles of the regions and their contributions to functional divergence. The swapped regions included the N-terminal equatorial, apical, and C-terminal equatorial regions between GroEL1 and GroEL2; the two highly similar intermediate regions were not included. The *groEL2* hybrids containing the N-terminal equatorial, apical or C-terminal equatorial region of *groEL1* were inserted into the genome of the *groEL1*-deletion mutant YL0301 using pSWU30, producing the region-swapped strains YL0903, YL0904 and YL0905, respectively. Similarly, the *groEL1* hybrids with a swapped N-terminal equatorial, apical or C-terminal equatorial region of *groEL2* were inserted into the genome of the *groEL2*-deletion mutant YL0302 to produce the mutant strains YL0908, YL0909 and YL0910, respectively (Figure 5A). Because the *groEL2* mutant displays defective cellular predation and the *groEL1* mutant displays deficient development and sporulation [15], region swapping was performed in YL0301 using the *groEL1* chimeras and YL0302 using the *groEL2* chimeras. Detailed descriptions of these mutants are listed in Tables S1 and S4 and Figure S4.

The development and predation phenotypes of the region-swapped mutants were assayed using the intact *groEL1*- and *groEL2*-complemented mutants as controls. The results showed that the developmental defect of the *groEL1*-deletion mutant was not reversed by GroEL2-equatorial-N_GroEL1_ (YL0903). The sporulation ability of YL0903 was approximately 20% of that of DK1622, which was the same as that of YL0301. However, the fruiting bodies of YL0904 (YL0301 complemented with GroEL2-apical_GroEL1_) were more similar to the fruiting bodies of the wild-type strain DK1622 than to the fruiting bodies of YL0301, and the sporulation ability also increased to 55%–65% of that of DK1622. The sporulation of the strain complemented with GroEL2-equatorial-C_GroEL1_ (YL0905) was 30%–40% of that of DK1622 (Figure 5B). In the predation experiments, the single swapped region in YL0908 did not noticeably improve the predation defects, which were similar to those of YL0906 (*P*>0.05). However, the YL0909 strain significantly recovered its predation ability, which was similar to that of YL0907. These two mutants spread to the edge of the *E. coli* colonies within 40–45 h. The predation defect was also improved to some extent in YL0910, which required 55–60 h to reach the edge of the *E. coli* mat (Figure 5C). Accordingly, the apical region and the C-terminal equatorial region determine the substrate preference, thus causing the functional divergence of the duplicated chaperonins with respect to development and predation; conversely, the N-terminal equatorial region has almost no effect.

In addition, we deleted the repeated GGM region (GGMGGMGGMGGMGGMGM) from GroEL1 in *M. xanthus* DK1622, producing the YL1001 mutant (Figure 5A). Compared with the wild-type DK1622, the mutant was markedly defective in development, and the sporulation ability of YL1001 was only 32.6% of that of DK1622 (Figure 5D). Furthermore, we swapped three of the six GGM repeats with YGGDDMDY in DK1622, the corresponding sequence in GroEL2, producing the YL1002 mutant. Similar to YL1001, YL1002 was also defective in development and exhibited a decreased sporulation ability (38.1% of that of DK1622) (Figure 5D). These results demonstrate that the GGM repeated region is necessary for GroEL1 to perform its normal functions in development.

### GroEL1 and GroEL2 substrates and their characteristics

To identify the proteins that interact with GroEL1 and GroEL2 in *M. xanthus* DK1622, immunoprecipitation assays were performed using the *groEL1*- and *groEL2*-deletion mutants (strains YL0301 and YL0302), and the bound proteins were subjected to mass spectral identification. Most of the non-specific substrates identified using two negative controls (see Methods) were ribosomal proteins (Table S5). This result was consistent with the results for *E. coli* [9]. After removing the non-specifically bound proteins, 151 and 114 proteins were found to be bound by GroEL1 and GroEL2, respectively. Of the bound proteins, 68 were bound to both GroEL1 and GroEL2 (GroEL1/2), whereas 83 and 46 proteins bound exclusively to GroEL1 and GroEL2, respectively (Table S5). Of the functionally annotated GroEL1/2 substrates (58/68, 85.3%), many had functions or predicted functions related to fundamental physiological cellular processes; examples of such substrates are succinyl coenzyme A synthetase and isocitrate dehydrogenase, two key enzymes of the citric acid cycle [29], [30]. This result is consistent with the fact that either the *groEL1* or *groEL2* gene could be deleted without affecting cellular growth but that the double deletion of *groEL1* and *groEL2* resulted in inviable cells [15]. However, except for PilA, no proteins involved in *M. xanthus* social behavior were found to bind to both GroEL1 and GroEL2. In contrast, of those annotated proteins that were exclusively bound by GroEL1 or GroEL2 (accounting for 75.9% and 76.1% of bound proteins, respectively), a considerable number are involved in the social behaviors of *M. xanthus* DK1622 (Table S5). For example, the *frz* signal transduction system is well known to play important roles in development process of *M. xanthus* DK1622 [31]. The frizzy aggregation protein FrzCD [31] is in the substrate list of GroEL1. Besides, Flp pilus assembly protein CpaB [18], sensor histidine kinase/response regulator CheA4 [32] and Type IV pilus secretin PilQ [33] were found to be specific substrates of GroEL1, whereas type IV pilus assembly ATPase PilB [34], gliding motility protein MglA [35], type IV pilus biogenesis protein PilM [36] and several proteins related to the biosynthesis of secondary metabolites were found to be exclusively bound by GroEL2. These results are consistent with the hypothesis that GroEL is an essential component and that the duplicated *groEL* genes evolved to participate in various complex physiological processes in *Myxococcus* cells.

**Figure 6:**
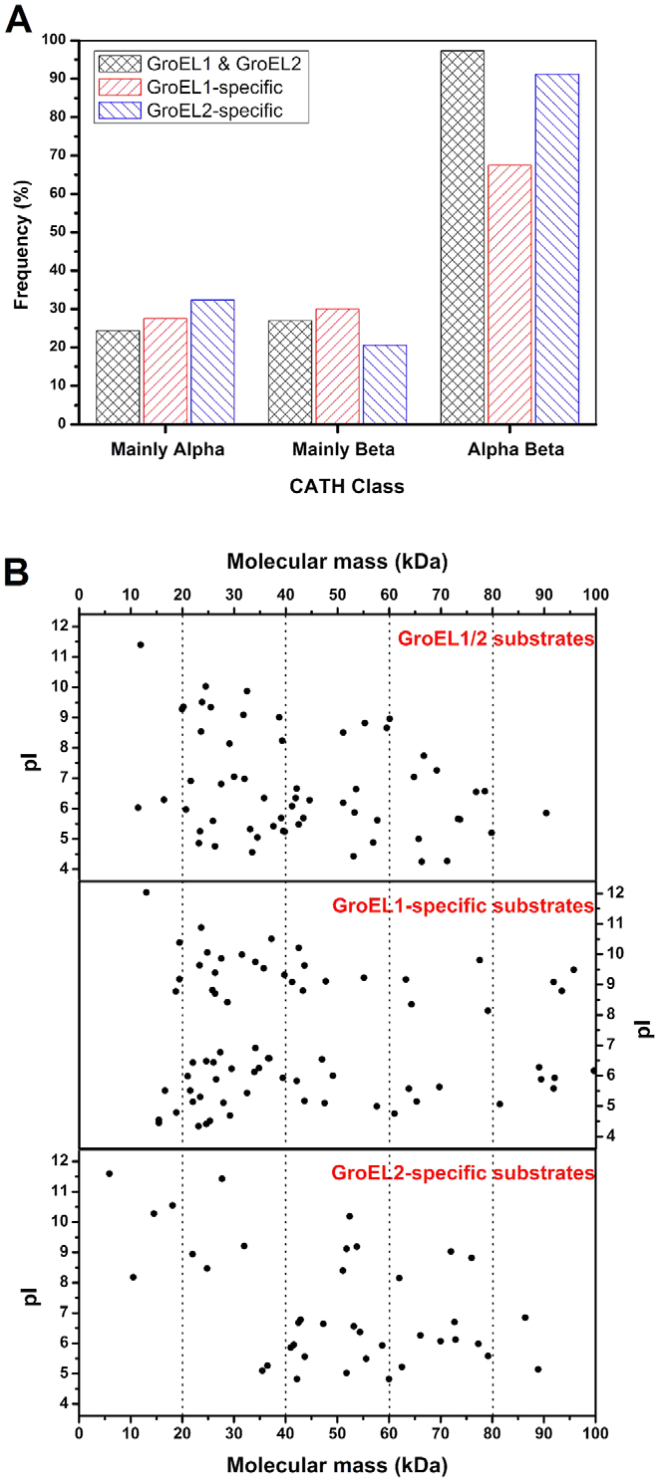
Substrate features of GroEL1 and GroEL2. (A) αβ-domain, (B) molecular weight distribution and pI distributions. The details of the substrate proteins of GroEL1 and GroEL2 are shown in Table S5.

The structural characteristics of the substrate proteins were further analyzed by comparing their secondary structures with the known protein domain classification database CATH [37]. After excluding the proteins that had low E-values (>0.001), we obtained 36 reliable secondary structures for the 68 identified GroEL1/2 substrates, 38 for the 83 GroEL1-specific substrates and 34 for the 46 GroEL2-specific substrates. It is known that proteins with β-sheets exposed to the hydrophobic surface and packed with the hydrophobic surfaces of α-helices (called the αβ domain) have high-affinity interactions with the apical region of GroEL and are normally present as substrates of GroELs [38]. As expected, most GroEL1/2 substrates (34 of 36) contain at least one αβ domain. It is interesting that, although 31 of the 34 (91.18%) GroEL2-specific substrates possess at least one αβ domain, only 27 of the 38 (71.05%) GroEL1-specific substrates contain an αβ domain (Figure 6A, Table S5).

Another interesting difference is the difference in the molecular sizes of the GroEL1 and GroEL2 substrates. According to the current model of GroEL [28], [39]–[45], substrate selection is heavily dependent upon the adaptation of a substrate molecule to the cavity volume of the GroEL chaperonin, which may change in response to GroEL sequence changes. Previous studies have shown that GroEL strongly prefers to act on proteins with a molecular weight ranging from 20 kDa to 60 kDa [46]. The average molecular weight of GroEL1-specific substrates was significantly smaller than that of GroEL2-specific substrates (Figure 6B; *P*<0.05). For example, while 51.8% (43 of 83) of the GroEL1-specific substrate proteins were less than 40 kDa, the molecular weights of only 21.7% (10 of 46) of the GroEL2-specific proteins were less than 40 kDa. A third important characteristic is the pI value of the substrate; there was no significant difference between the GroEL1 and GroEL2 substrates with respect to pI (Figure 6B; *P*>0.05).

## Discussion

Duplication is a major source of new genes and is equally important in Bacteria, Archaea and Eukarya [47], [48]. The duplication of the GroEL chaperonin gene has occurred in many different bacterial cells as part of the evolution of complexity [49]. *M. xanthus* DK1622 is well known for its complex multicellular behaviors [16], [17], and this strain possesses a large genome (9.14 Mb) in which there are many duplicated genes, including two copies of *groEL* [18]. In addition to participating in fundamental processes involved in cellular growth, the two duplicated *groEL* genes have been demonstrated to play distinct roles in heat-shock responses, development and predation in DK1622 [15]. The results described in this report show that the divergent functions of GroEL1 and GroEL2 in various physiological processes result from different mechanisms. The *groEL* expression level is the key reason for the difference in the heat-shock response after the deletion of *groEL1* or *groEL2*, suggesting that the duplicated *groEL* genes in *Myxococcus* have similar functions in cell survival. These functions are most likely similar to their fundamental function in cell growth at normal temperatures. Either of the two *groEL* genes can be deleted without significantly affecting cell growth, but at least one *groEL* gene is required for cell survival [15]. In contrast, the functional divergence of the duplicated GroELs with respect to their roles in development and predation processes reflects in their substrate specificity, which has been suggested to evolutionarily relate to the unusual social behavior of *Myxococcus*. The co-substrates of GroEL1 and GroEL2 have consistently been shown to be essential cellular components, but the duplicated GroEL chaperonins have also evolved their own substrate preferences related to late-appearing cellular processes, such as social behaviors and PKS/NRPS biosynthesis. The evolutionary models for the functional divergence of duplicated genes are likely to include neofunctionalization, subfunctionalization, or a combination of thereof [20], [47], [50]. Thus, the functional divergence of the duplicated *groELs* in *M. xanthus* is likely a combination of neofunctionalization and subfunctionalization, *i.e.*, the subneofunctionalization model [50].

Although extensive studies have demonstrated that the duplicated *groEL* genes play distinct roles in different cellular physiological processes [10]–[15], an understanding of the mechanisms involved in their functional divergence will provide insight into bacterial evolution. The GroEL proteins have been divided into five regions based on structural characteristics and sequence conservation [28]. Previous studies have shown that GroEL chimeras bearing equatorial or apical regions exchanged between *M. tuberculosis* and *E. coli* retained the normal chaperonin functions of GroEL [51]. Bioinformatics analyses indicate that the duplicated GroELs from different bacteria share similar characteristics: the N- and C-intermediate regions are highly conserved, suggesting that these regions have essential functions in maintaining the functional structure of GroELs, and the apical, N-terminal and C-terminal regions are much more flexible, suggesting their possible roles in functional divergence. The region-swapping experiments indicated that the functional divergence of the duplicated GroELs in *M. xanthus* was caused by the apical and C-terminal regions. The GGM repeat at the tail of the GroEL1 C-terminal region, which is similar to that of *E. coli* GroEL, is important for the distinct functions of GroEL1 in development and sporulation. These results are consistent with the positions of these two regions in the GroEL oligomeric complex, *i.e.*, the apical region is at the opening through which substrates enter the central cavity, and the C-terminal equatorial region is at the bottom of the cavity [43]. However, it remains unclear whether region swapping has effects on *in vivo* chaperonin functions. To address the question, we assayed the survival rates of the mutants YL0903, YL0904 and YL0905 in response to heat shock and found that all the mutants rescued the lethality of heat-shock observed for YL0301. However, the survival rates of YL0903, YL0904, and YL0905 were low compared with the strains complemented with an intact *groEL* gene (Figure S5). This result suggests that the region-swapped GroELs function in *M. xanthus* cells but that these functions were affected, at least at non-permissive temperatures. It is still unclear whether the chimeras interact with intact GroELs and whether the *in vivo* functions of the chimeras result from mixed GroEL complexes. Furthermore, although the protein substrates of the *M. xanthus* GroELs were consistent with those of the single *E. coli* GroEL with regard to their secondary structural features [9], the substrate spectra varied significantly. This variation is most likely due to the low level of sequence similarity between the *E. coli* GroEL and the *M. xanthus* GroELs (*E. coli* GroEL is 67.3% and 65.2% similar to *M. xanthus* GroEL1 and GroEL2, respectively) and to the difference in the protein substrates between these two bacteria. Therefore, there are many questions related to the GroEL chaperonins and their functional divergence that need to be addressed.

## Methods

### Cultures, plasmids, and growth conditions

The strains and plasmids used in this study are listed in Table S1. For the growth assays, the *M. xanthus* strains were cultivated in the Casitone-based nutrient-rich CTT medium [52]. The *E. coli* strains were routinely grown on Luria-Bertani (LB) agar or in LB broth. *E. coli* was grown at 37°C, whereas the *Myxococcus* strains were incubated at 30°C. When required, 40 µg/ml of kanamycin (Km) and 10 µg/ml of tetracycline (Tet) (Sigma) were added to the medium.

### Expression analysis of *groEL* genes during heat shock, predation, and development

The *groEL* expression levels during heat shock and liquid predation were analyzed using quantitative real-time PCR. *M. xanthus* DK1622 and other mutants were harvested after 18 h and exposed to 42°C for 1 h. The RNA was extracted immediately using a total RNA extraction kit following the manufacturer's instructions (Promega). Contaminating DNA was removed with a DNAfree kit (Ambion). The purified RNA was transcribed to yield cDNA, which was stored at −70°C. The quantitative real-time PCR was performed using a Bio-Rad sequence detection system with 250 nM primers, 10 µl of SYBR Green PCR Master Mix (Bio-Rad), 7 µl of RNase-free water, and 2 µl of cDNA template. The PCR was performed for 3 min at 95°C, followed by 40 cycles of 30 s at 95°C, 30 s at 59°C, and 15 s at 72°C. The 16S rRNA was used as a normalization signal. Calibration curves (*groEL1*, *groEL2*, and 16S RNA) were generated using 10-fold dilutions of *M. xanthus* DK1622 genomic DNA. The following pairs of forward and reverse primer pairs were used: *groEL1*, 5′-CACCGAGACGGAGATGAAGG-3′ and 5′-TGAGGCAGCGGATGTAGGC-3′; *groEL2*, 5′-ATCCGCACGCAGATTGAC-3′ and 5′-GCfCTTCTTCTCCTTCATCTCC-3′; and 16S rRNA, 5′-CGCCGTAAACGATGAGAA-3′ and 5′-TTGCGTCGAATTAAACCAC-3′. The *groEL* expression levels during predation were analyzed using quantitative real-time PCR. The strains were cultured for 50 h in medium containing casein as a substrate instead of hydrolyzed proteins, and the RNA was extracted immediately. The method and the primers used were the same as those described above. The *groEL* expression level during different developmental stages was analyzed by measuring the β-galactosidase activity, as described by Li *et al.* [15], [53], with minor modifications. The cells were broken using a Mini-Beadbeater (BioSpec) at a speed of 2500 rpm. The β-galactosidase activity was determined using o-nitrophenyl-β-galactopyranoside (Sigma), and the samples were analyzed at 420 nm. The total protein concentration was determined using the bicinchoninic acid protein assay (Pierce). The specific activity was calculated as follows: specific activity = 213×A420/(sample volume×protein concentration×reaction time) [15], [54].

### Development assays

*M. xanthus* cells were harvested at mid-logarithmic phase and suspended to a final density of 5×10^9^ cells/ml in TPM buffer. Aliquots (10 µl) were spotted onto TPM agar, and the plates were cultivated at 30°C and observed every 24 h to monitor the formation of fruiting bodies. The sporulation rate was measured after 5 days as previously described. The assays were performed at least three times [15].

### Predation assays

The predation assays were performed according to the method used in a previous study [15]. *E. coli* and *M. xanthus* cultures were harvested at mid-logarithmic phase and washed three times with 10 mM MOPS buffer (pH 7.6). The final cell densities of the cultures were 5×10^9^ cells/ml for *M. xanthus* and 1×10^11^ cells/ml for *E. coli*. Then, 50 µl of *E. coli* was pipetted onto a plate to form a 1-cm-diameter colony, and 2 µl of *M. xanthus* was added to the center of the *E. coli* colony, with an inoculation diameter of 0.15 cm. The assay was repeated at least three times. The plates were incubated at 30°C for 6 days, during which time the size of the *M. xanthus* growth area was recorded every 12 h. The predation ability of *M. xanthus* was reported as the time required for *M. xanthus* to spread to the edge of the *E. coli* colonies.

### Heat-shock assays

*M. xanthus* cultures were harvested as described above. The cells were heat shocked for 30 min at 42°C, serially diluted and plated on CTT agar. After 6 days incubation, the CFUs were calculated [15].

### Evolutionary analysis

The *groEL* gene sequences from ten genome-sequenced bacterial strains were retrieved from the NCBI database (Table S2), and the amino acid sequences were aligned using the protein sequence alignment program in CLUSTALW [55]. A maximum likelihood tree was constructed using MEGA5 [56]. The Ka/Ks values among orthologous *groEL* genes or among paralogous *groEL* genes were calculated using KaKs_Calculator 1.2 [57] with the NG, MLWL and MLPB models [58], [59].

### Vector construction for the region swapping of *groELs* and mutant identification

The region-swapping assay was conducted according to a previously published method [51]. The regions responsible for the developmental defects of YL0301 and the predation defects of YL0302 were investigated by incorporating single *groEL* regions into YL0301 or YL0302. The complementation mutants were constructed with the site-specific integration plasmid pSWU30. The apical region of *groEL1* was inserted into YL0301 to obtain YL0904 (YL0301::pSWU- *groEL2*-apical*_groEL1_*). Briefly (Figure S4), 0.5 kb of the upstream sequence and the N-terminal region (bp 1–597) of *groEL2* and the C-terminal (597 to the end) of *groEL1* were amplified by PCR. The two fragments were spliced by fusion PCR, digested with XbaI and BamHI, and ligated into pSWU30 digested with XbaI and BamHI. The plasmid was transferred to *E. coli* λ-pir cells, and the plasmid DNA was extracted from the Tet-resistant transformants using the eZNA Plasmid Mini Kit I (Omega Bio-Tek) according to the manufacturer's instructions. The correct plasmid was used as the template in the second round of fusion PCR. The plasmid containing the correct sequence was transferred by electroporation into YL0301, and individual Tet-resistant colonies were screened. The mutant phenotypes were observed to determine the effects of the apical region on development. The same method was used to replace other regions. The primers used are listed in Table S4.

### Vector construction for the deletion of the GGM region and the truncation of *groEL1*

The GGM region deletion mutants were constructed using the positive-negative KG cassettes described by Ueki *et al.* Briefly, the upstream sequence (before the GGM sequence) and the downstream sequence (after the GGM sequence) were amplified by PCR. The two fragments were fused to the XbaI restriction site to construct homologous fragments with in-frame deletions. These homologous fragments were ligated into SmaI-digested pBJ113. The resulting plasmid containing the correct sequence was transferred by electroporation into DK1622. The second round of screening was then performed on CTT plates containing 1% galactose (Sigma). The deletion mutants that grew on galactose but were sensitive to kanamycin were identified and verified by PCR and sequencing. The GGM sequence-swapping mutants were constructed in a similar manner. The upstream sequence (739–1623 of *groEL1*+the DNA sequence corresponding to the last eight amino acids in *groEL2*) and the downstream sequence (the DNA sequence corresponding to the last eight amino acids in *groEL2*+815 bp downstream of *groEL1*) were PCR amplified. The two fragments were fused to the XbaI site to construct in-frame deletion fragments that were ligated into SmaI-digested pBJ113. The resulting plasmid was subjected to two rounds of screening to obtain the GGM-swapping mutant. The phenotypes of the mutants were observed to determine the effects of the GGM repeat region on development. The primers used are listed in Table S6.

### Immunoprecipitation assay

*groEL1*-ko and *groEL2*-ko mutants were resuspended in Tris buffer (50 mM Tris, 150 mM NaCl, 5 mM EDTA, and 20 µl PMSF) after cultivation and washed three times. The cells were lysed using a high-pressure homogenizer. Aliquots of 50 µl of protein A/G beads were added to 10 ml of supernatant solution to remove the proteins that non-specifically bound to the beads. An anti-GroEL antibody (3 mg/ml) was added, and the solution was shaken at low speed at 4°C. Aliquots of 100 µl of protein A/G beads were mixed with 50 ml of solution and incubated for another 2 h. The beads bound by the GroEL substrates were washed with Tris buffer three times, and the beads and proteins were separated with lysis buffer. The substrates of GroELs were identified by high-pressure liquid chromatography-tandem mass spectrometry (LC-MS/MS) by Shanghai Zhongke Biotech Company. A negative control assay was performed with DK1622 using the same protocol in the absence of the antibody against GroEL to exclude non-specific binding between the beads and proteins [38]. To exclude non-specific binding between the antibody and proteins and to exclude non-specific binding between GroEL and proteins after cell lysis, another negative control was performed by adding 0.1% SDS to the lysis buffer to separate GroEL from its substrates [60]. The solution was diluted 50 fold after cell lysis and the addition of extra GroEL1 and GroEL2 protein. The antibody against GroEL was then added to identify non-specifically bound proteins [38], [60].

## Supporting Information

Figure S1 The correlation between GroELs' expression level and relative suvival under heat shock condition.
(TIF)

Figure S2 Expression of *groEL* genes in the process of liquid feeding assay. The values for each *groEL* gene are shown as relative levels to the expression level of *groEL1* in DK1622, which is defined as 100%.
(TIF)

Figure S3 The five regions of the amino acid sequences of the two *groEL* genes in *Myxococcus xanthus* DK1622. The regions (from N- to C-terminus) are the N-terminal equatorial region, intermediate region, apical region, intermediate region, C-terminal equatorial region.
(TIF)

Figure S4 Schematic diagram of fusion-PCR for the region swapping experiments, using YL0904 as a demonstration.
(TIF)

Figure S5 Relative survival of region-swapping and GGM deletion mutants under heat shock condition.
(TIF)

Table S1 Bacterial strains and plasmids used in this study.
(PDF)

Table S2 Sequence information used by evolutionary analysis.
(PDF)

Table S3 Original data of Ka/Ks values.
(PDF)

Table S4 List of primers for fusion-PCR for domain swapping assay.
(PDF)

Table S5 The information of the identified substrates bound by GroEL1 and/or GroEL2. S5-1, Substrates of both GroEL1 and GroEL2. S5-2, Specific substrates of GroEL1. S5-3, Specific substrates of GroEL2. S5-4, Non-specific substrates of GroEL.
(PDF)

Table S6 List of primers for GGM region deletion and shorten.
(PDF)
