# Supplementary figures and images for: Mechanisms Involved in the Functional Divergence of Duplicated GroEL Chaperonins in Myxococcus xanthus DK1622
Source: PLoS Genet. 2013 Feb 21;9(2):e1003306. doi: 10.1371/journal.pgen.1003306 (PMC3578752; doi:10.1371/journal.pgen.1003306)

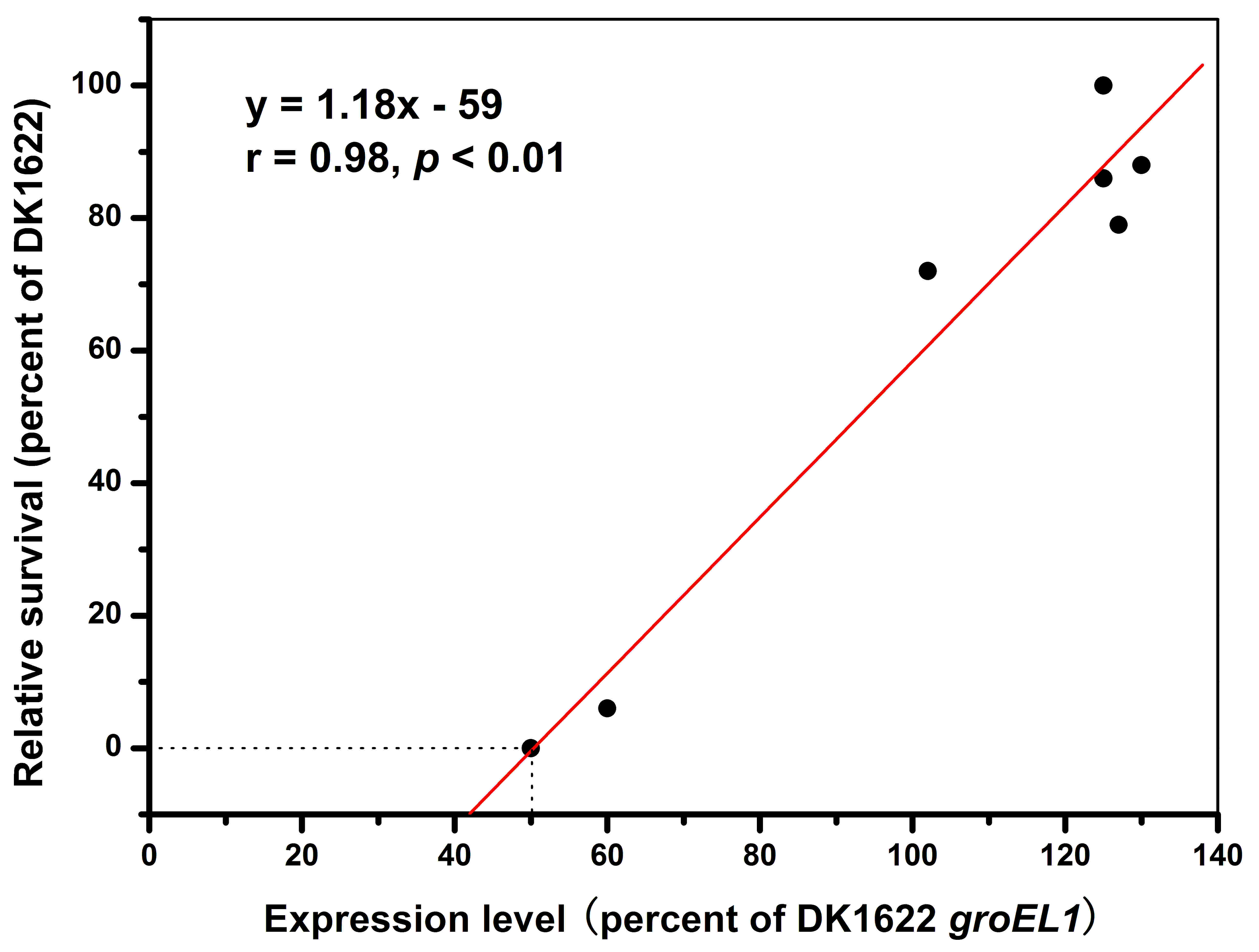

Supplement: Figure S1 — The correlation between GroELs' expression level and relative suvival under heat shock condition. (TIF) [file pgen.1003306.s001.tif]

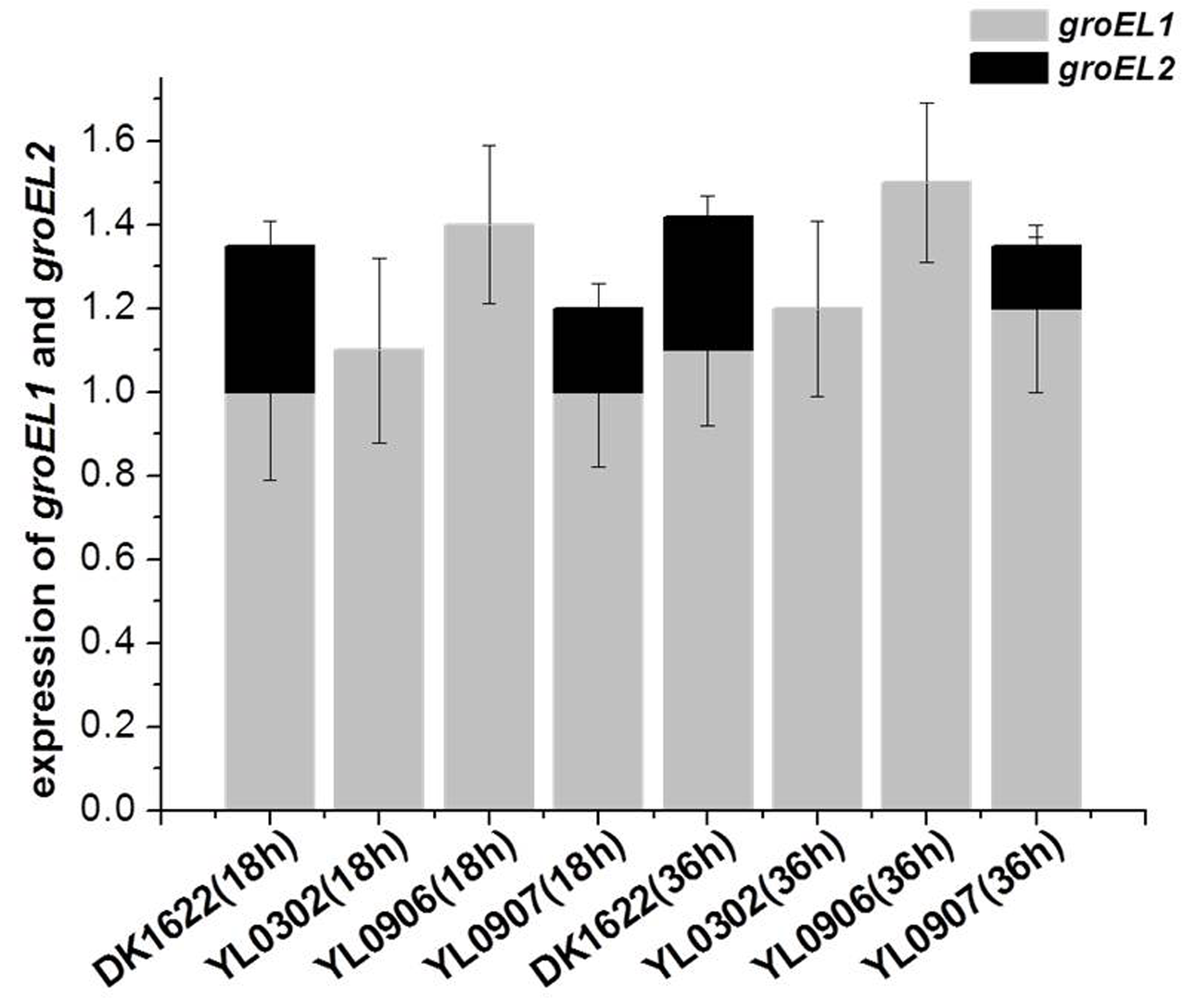

Supplement: Figure S2 — Expression of groEL genes in the process of liquid feeding assay. The values for each groEL gene are shown as relative levels to the expression level of groEL1 in DK1622, which is defined as 100%. (TIF) [file pgen.1003306.s002.tif]

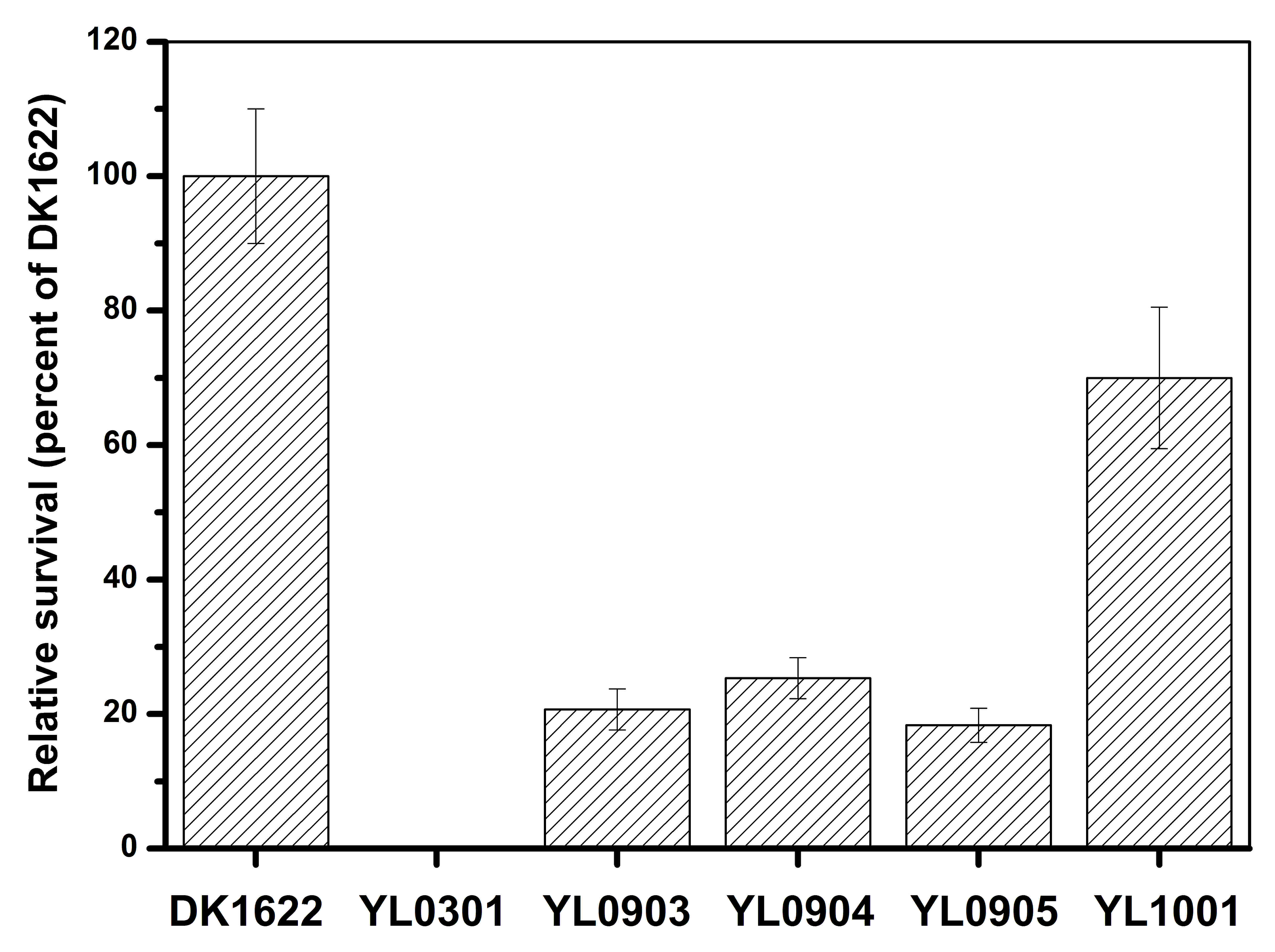

Supplement: Figure S4 — Schematic diagram of fusion-PCR for the region swapping experiments, using YL0904 as a demonstration. (TIF) [file pgen.1003306.s004.tif]

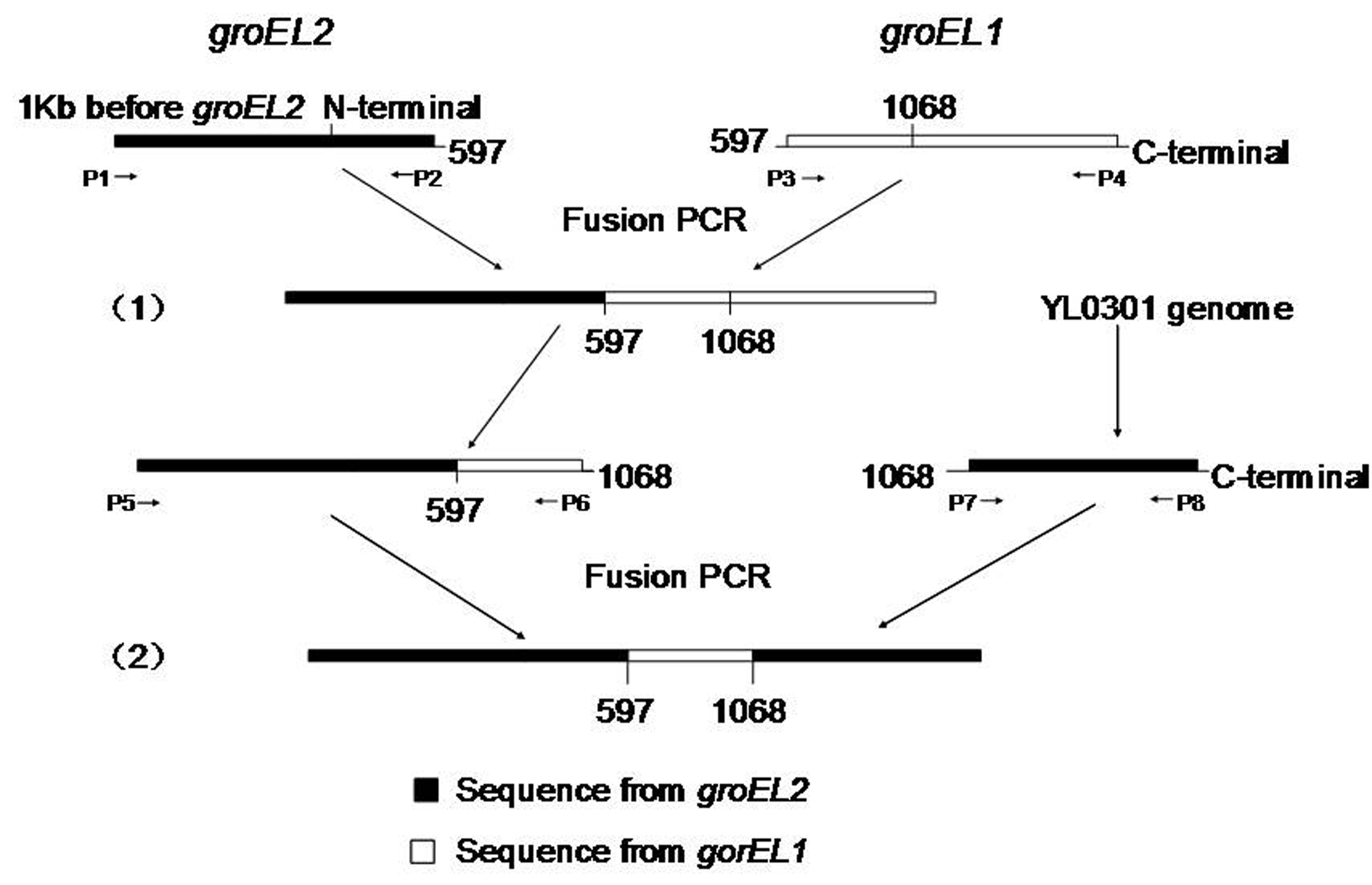

Supplement: Figure S5 — Relative survival of region-swapping and GGM deletion mutants under heat shock condition. (TIF) [file pgen.1003306.s005.tif]
