# Supplementary material for: Mechanisms Involved in the Functional Divergence of Duplicated GroEL Chaperonins in Myxococcus xanthus DK1622
Source: PLoS Genet. 2013 Feb 21;9(2):e1003306. doi: 10.1371/journal.pgen.1003306 (PMC3578752; doi:10.1371/journal.pgen.1003306)
Supplement: Table S1 — Bacterial strains and plasmids used in this study. (PDF) [file pgen.1003306.s006.pdf]

Table S1. Bacterial strains and plasmids used in this study

Strain and plasmid	Genotype or description	Source or reference
**Strains**		
M. Xanthus DK1622	Wild-type strain	Kaiser *et al.* (1979)
YL0301	DK1622 Δ*MXAN_4895* (*groEL1* deletion)	Lj *et al.*(2010)
YL0302	DK1622 Δ*MXAN_4467* (*groEL2* deletion)	Lj *et al.* (2010)
YL0901	YL0301:: pSWU-*groEL1* (*groEL1* integrated at *attB* site)	This study
YL0902	YL0301:: pSWU-*groEL2* (*groEL2* integrated at *attB* site)	This study
YL0903	YL0301:: pSWU-*groEL2*-equatorial-N_{groEL1}	This study
YL0904	YL0301:: pSWU-*groEL2* –$apical_{groEL1}$	This study
YL0905	YL0301:: pSWU-*groEL2*-equatorial-C_{groEL1}	This study
YL0906	YL0302:: pSWU-*groEL1*	This study
YL0907	YL0302:: pSWU-*groEL2*	This study
YL0908	YL0302:: pSWU-*groEL1*-equatorial-N_{groEL2}	This study
YL0909	YL0302:: pSWU-*groEL1*-$apical_{groEL2A}$	This study
YL0910	YL0302:: pSWU-*groEL1*-equatorial-C_{groEL2}	This study
YL0911	DK1622:: pZC4895 (*lacZ* fused to *groEL1*)	This study
YL0912	DK1622:: pZC4467 (*lacZ* fused to *groEL2*)	This study
YL0913	YL0301:: pZC4467 (*lacZ* fused to *groEL2*)	This study
YL0914	YL0901:: pZC4895 (*lacZ* fused to *groEL1*)	This study
YL0915	YL0901:: pZC4467 (*lacZ* fused to *groEL2*)	This study
YL0916	YL0902:: pZC4467 (*lacZ* fused to *groEL2*)	This study
YL1001	DK1622 ΔGGM repeat of GroEL1 (GGM repreat deletion)	This study
YL1002	DK1622 Δthe last 3 GGM of GroEL1 (the last 3 GGM substitution)	This study
E. coli		
DH5α(λpir)	f80 *lacZ_M15 _lacU169 recA1 endA1 hsdR17 supE44 thi-1 gyrA relA1λpir*	H. B. Kaplan, University of Texas

BL21(DE3)	F^- *ompT hsdS$_B$* ($r_B^- m_B^-$)*gal dcm*	Stratagene
XL1-Blue MR	*Δ(mcrA)183Δ(mcrCB-hsdSMR-mrr)173 endA1 supE44 thi-1 recA1 gyrA96 relA1 lac*	Stratagene
**Plasmids**		
pSWU30	Site-specific integration vector with Mx8 attB integration site; Tet^r	Tâm Mignot, CNRS (Centre national de la recherche scientifique)
pSWU-*groEL1*	*groEL1* was ligated with pSWU30. Tet^r	This study
pSWU-*groEL2*	*groEL2* was ligated with pSWU30. Tet^r	This study
pSWU-*groEL2*-equatorial-N_{groEL1}	Equatorial-N region belong to GroEL1 and the other regions belong to GroEL2. The two fragments were fused and the fused fragment was ligated with pSWU30. Tet^r	This study
pSWU-*groEL1*-equatorial-N_{groEL2}	Equatorial-N region belong to GroEL2 and the other region belong to GroEL1. The two fragments were fused and the fused fragment was ligated with pSWU30. Tet^r	This study
pSWU-*groEL2*-apical$_{groEL1}$	Apical region belong to GroEL1 and the other region belong to GroEL2. the two fragments were fused and the fused fragment was ligated with pSWU30. Tet^r	This study
pSWU-*groEL1*-apical$_{groEL2}$	Apical region belong to GroEL2 and the other region belong to GroEL1. the two fragments were fused and the fused fragment was ligated with pSWU30. Tet^r	This study
pSWU-*groEL2*-equtorial-C_{groEL1}	Equatorial-C region belong to GroEL1 and the other region belong to GroEL2. The two fragments were fused and the fused fragment was ligated with pSWU30. Tet^r	This study
pSWU-*groEL1*-equtorial-C_{groEL2}	Equatorial-C region belong to GroEL2 and the other region belong to GroEL1. The two fragments were fused and the fused fragment was ligated with pSWU30. Tet^r	This study
pZCY11	1.56-kb HindIII/EcoRI fragment from pSL1180-Km, blunted with Klenow fragment and ligated with 4.57-kb T4 polymerase-blunted KpnI fragment from pMiniHimar1-*lacZ*, Km^r *lacZ*	Lj *et al.*(2010)
pZC4895	735-bp fragment of DK1622 *MXAN_4895*, containing a stop codon, inserted into SpeI/KpnI sites of pZCY11, Km^r *lacZ*	This study
pZC4467	601-bp fragment of DK1622 *MXAN_4467*, containing a stop codon, inserted into SpeI/KpnI sites of pZCY11, Km^r *lacZ*	This study
