# Supplementary material for: Mechanisms Involved in the Functional Divergence of Duplicated GroEL Chaperonins in Myxococcus xanthus DK1622
Source: PLoS Genet. 2013 Feb 21;9(2):e1003306. doi: 10.1371/journal.pgen.1003306 (PMC3578752; doi:10.1371/journal.pgen.1003306)
Supplement: Table S2 — Sequence information used by evolutionary analysis. (PDF) [file pgen.1003306.s007.pdf]

Table S2. Sequence information used by evolutionary analysis

Species	Gene name	Locus tag	Protein product	Length (bp)
Myxococcus xanthus DK 1622	*groEL2*	MXAN_4467	YP_632638.1	1644
	groEL1	MXAN_4895	YP_633052.1	1650
Myxococcus fulvus HW-1	*groEL2*	LILAB_30470	YP_004669053.1	1641
	groEL1	LILAB_32445	YP_004669448.1	1650
Anaeromyxobacter dehalogenans 2cp-1	*groEL2*	A2cp1_1565	YP_002491975.1	1644
	groEL1	A2cp1_3735	YP_002494127.1	1644
Anaeromyxobacter sp. fw109-5	*groEL2*	Anae109_1472	YP_001378663.1	1644
	groEL1	Anae109_3715	YP_001380878.1	1644
Mycobacterium tuberculosis H37Rv	*groEL2*	Rv0440	NP_214954.1	1623
	groEL1	Rv3417c	NP_217934.1	1620
Mycobacterium smegmatis str. MC^2 155 *	*groEL2*	MSMEG_0880	YP_885283.1	1626
	groEL1	MSMEG_1583	YP_885962.1	1623
Corynebacterium glutamicum ATCC 13032	*groEL1*	NCgl0573	NP_599834.1	1617
	groEL2	NCgl2621	NP_601912.1	1647
Synechocystis sp. PCC 6803	*groEL1*	slr2076	NP_440731.1	1626
	groEL2	sll0416	NP_442170.1	1659
Synechococcus elongatus PCC 7942	*groEL2*	Synpcc7942_0685	YP_399704.1	1668
	groEL1	Synpcc7942_2313	YP_401330.1	1635
Thermosynechococcus elongatus BP-1	*groEL1*	tll0185	NP_680976.1	1638
	groEL2	tlr1412	NP_682202.1	1632
Escherichia coli str. K-12 substr. MG1655	*groEL*	b4143	NP_418567.1	1647

**M. smegmatis* also encodes a third GroEL (MSMEG_1978), it is more distantly related to GroEL1 and GroEL2.
