# Supplementary material for: Mechanisms Involved in the Functional Divergence of Duplicated GroEL Chaperonins in Myxococcus xanthus DK1622
Source: PLoS Genet. 2013 Feb 21;9(2):e1003306. doi: 10.1371/journal.pgen.1003306 (PMC3578752; doi:10.1371/journal.pgen.1003306)
Supplement: Table S3 — Original data of Ka/Ks values. (PDF) [file pgen.1003306.s008.pdf]

Table S3. Original data of Ka/Ks values

Sequence pairs*			Method	Ka	Ks	Ka/Ks
M. xanthus	*M. fulvus*	*groEL1*	NG	0.010	0.069	0.143
			MLWL	0.010	0.075	0.129
			MLPB	0.010	0.075	0.131
		groEL2	NG	0.014	0.039	0.366
			MLWL	0.014	0.040	0.349
			MLPB	0.015	0.042	0.344
M. xanthus	*A. dehalogenans*	*groEL1*	NG	0.103	0.364	0.283
			MLWL	0.096	0.510	0.188
			MLPB	0.101	0.512	0.197
		groEL2	NG	0.148	0.399	0.370
			MLWL	0.138	0.626	0.220
			MLPB	0.146	0.630	0.232
M. xanthus	*Anaeromyxobacter* sp.	*groEL1*	NG	0.117	0.407	0.287
			MLWL	0.109	0.565	0.193
			MLPB	0.113	0.557	0.204
		groEL2	NG	0.139	0.440	0.315
			MLWL	0.130	0.690	0.188
			MLPB	0.137	0.699	0.196
M. tuberculosis	*M. smegmatis*	*groEL1*	NG	0.130	0.828	0.157
			MLWL	0.128	0.865	0.148
			MLPB	0.131	0.901	0.146
		groEL2	NG	0.039	0.421	0.093
			MLWL	0.038	0.444	0.086
			MLPB	0.038	0.437	0.086
M. tuberculosis	*C. glutamicum*	*groEL1*	NG	0.328	1.891	0.173
			MLWL	0.323	2.013	0.161
			MLPB	0.329	1.965	0.168
		groEL2	NG	0.126	1.210	0.104
			MLWL	0.125	1.446	0.086
			MLPB	0.122	1.286	0.095
M. smegmatis	*C. glutamicum*	*groEL1*	NG	0.277	1.467	0.189
			MLWL	0.272	1.599	0.170
			MLPB	0.274	1.454	0.189
		groEL2	NG	0.108	1.055	0.102
			MLWL	0.108	1.562	0.069
			MLPB	0.104	1.343	0.077
Synechocystis sp.	*S. elongatus*	*groEL1*	NG	0.101	1.575	0.064
			MLWL	0.101	1.478	0.069
			MLPB	0.100	1.317	0.076
		groEL2	NG	0.250	1.806	0.138

			MLWL	0.247	1.667	0.148
			MLPB	0.251	1.698	0.148
Synechocystis sp.	*T. elongatus*	*groEL1*	NG	0.092	1.426	0.065
			MLWL	0.094	1.195	0.078
			MLPB	0.093	1.112	0.084
		groEL2	NG	0.239	1.823	0.131
			MLWL	0.240	1.678	0.143
			MLPB	0.240	1.547	0.155
S. elongatus	*T. elongatus*	*groEL1*	NG	0.085	1.566	0.055
			MLWL	0.085	1.377	0.062
			MLPB	0.084	1.268	0.066
		groEL2	NG	0.239	1.695	0.141
			MLWL	0.237	1.572	0.151
			MLPB	0.241	1.567	0.154

*Because of low identity at the C-terimial of each *groEL* gene, we removed the last 90bp in *E. coli* and corresponding sequence in the other *groEL* genes in this part.
