# Supplementary material for: Mechanisms Involved in the Functional Divergence of Duplicated GroEL Chaperonins in Myxococcus xanthus DK1622
Source: PLoS Genet. 2013 Feb 21;9(2):e1003306. doi: 10.1371/journal.pgen.1003306 (PMC3578752; doi:10.1371/journal.pgen.1003306)
Supplement: Table S4 — List of primers for fusion-PCR for domain swapping assay. (PDF) [file pgen.1003306.s009.pdf]

Table S4. List of primers for fusion-PCR for domain swapping assay

Oligonucleotide Primers		Oligonucleotide sequence (5'→3')	Reference
Apical region	primer for the research of apical region of *groEL1:*	P1: GCTCTAGAGAACTCTGCACGATGCTCTCTC	PCR amplification of upstream sequence (0.5kb) + the sequence of N-terminal (1-597bp) of *groEL2*
		P2: GGTCCGTCACGAAGTACGGGGACAGGTAGCCGCGGTCGA ACTGCATGCCC	
		P3: GGGCATGCAGTTCGACCGCGGCTACCTGTCCCCGTACTTC GTGACGGACC	PCR amplification of C-terminal (597 bp to the end) of *groEL1*
		P4: CGGGATCCCTACATGCCCATACCGCCCATACCG	
		P5: GCTCTAGAGAACTCTGCACGATGCTCTCTC	PCR amplification of upstream sequence(0.5kb)+ the sequence of N-terminal (1-597bp) of *groEL2* +the sequence(597-1068bp) of *groEL1*
		P6: GGAGCTTCTCGCGGTCGTAGTCGCTGGAGGTCTCCTCGAT CTGGGCGCGG	
		P7: CCGCGCCCAGATCGAGGAGACCTCCAGCGACTACGACCG CGAGAAGCTCC	PCR amplification of C-terminal (1068bp to the end) of *groEL2*
		P8: CGGGATCCTCAGTAGTCCATGTCGTCGCCGC	
	primer for the research of apical region of *groEL2*	P1: GCTCTAGAACACCTCCCGTCATTCCCCG	PCR amplification of upstream sequence(0.5kb)+ the sequence of N-terminal (1-597bp) of *groEL1*
		P2: GGTTGGTCACGAAGTACGGGGACACGTAGCCGCGGTCGA ACTGCATGCCT	
		P3: AGGCATGCAGTTCGACCGCGGCTACGTGTCCCCGTACTTC GTGACCAACC	PCR amplification of C-terminal (597 bp to the end) of *groEL2*
		P4: CGGGATCCTCAGTAGTCCATGTCGTCGCCGC	
		P5: GCTCTAGAACACCTCCCGTCATTCCCCG	PCR amplification of upstream sequence (0.5kb)+ the sequence of N-terminal (1-597bp) of *groEL1*+ the sequence (597-1068bp) of *groEL2*
		P6: GGAGCTTCTCGCGGTCGTAGTCGCTGGTGACGGAGTCAA TCTGCGTGCGG	
		P7: CCGCACGCAGATTGACTCCGTCACCAGCGACTACGACCG CGAGAAGCTCC	PCR amplification of C-terminal (1068bp to the end) of *groEL1*
		P8: CGGGATCCCTACATGCCCATACCGCCCATACCG	

Equatorial-N region	primer for the research of equatorial-N region of *groEL1*	P1: GCTCTAGAGAACTCTGCACGATGCTCTCTC	PCR amplification of upstream sequence (0.5kb) of *groEL2*
		P2: CGTCGAAAAGAATGTCCTTCGCCATCGCAACCTCCTGAAT TCACTGGGGG	
		P3: CCCCCAGTGAATTCAGGAGGTTGCGATGGCGAAGGACAT TCTTTTCGACG	PCR amplification the sequence of *groEL1*
		P4: CGGGATCCCTACATGCCCATACCGCCCATACCG	
		P5: GCTCTAGAGAACTCTGCACGATGCTCTCTC	PCR amplification of upstream sequence (0.5kb) of *groEL2*+the sequence(1-438bp) of *groEL1*
		P6: CGTTGGCGGAGATGGTCCCCACCTGGGCAATCTCCTTCTT GTCCTTGGTC	
		P7: GACCAAGGACAAGAAGGAGATTGCCCAGGTGGGGACCA TCTCCGCCAACG	PCR amplification of C-terminal (438bp to the end) of *groEL2*
		P8: CGGGATCCTCAGTAGTCCATGTCGTCGCCGC	
	primer for the research of equatorial-N region of *groEL2*	P1: GCTCTAGAACACCTCCCGTCATTCCCCG	PCR amplification of upstream sequence (0.5kb) of *groEL1*
		P2: GGAAGAAAATTTCCTTCGCTGCCATTTGGATGGTTCCTTG AAGGAAGAGT	
		P3: ACTCTTCCTTCAAGGAACCATCCAAATGGCAGCGAAGGA AATTTTCTTCC	PCR amplification the sequence of *groEL2*
		P4: CGGGATCCTCAGTAGTCCATGTCGTCGCCGC	
		P5: GCTCTAGAACACCTCCCGTCATTCCCCG	PCR amplification of upstream sequence (0.5kb) of *groEL1*+the sequence (1-438bp) of *groEL2*
		P6: CGTTGGCGGAGATGGTACCGACCTGGGTGATGGCCTTCTT GTCGGCGGTG	
		P7: CACCGCCGACAAGAAGGCCATCACCCAGGTCGGTACCAT CTCGCCAACG	PCR amplification of C-terminal (438bp to the end) of *groEL1*
		P8: CGGGATCCCTACATGCCCATACCGCCCATACCG	
Equatorial-C region	primer for the research of equatorial-C region of *groEL1*	p1: GCTCTAGAGAACTCTGCACGATGCTCTCTC	PCR amplification of upstream sequence (0.5kb)+the sequence of N-terminal (1-1254bp) of *groEL2*
		p2: CGTCCAGCGCCTTGAGGCAGCGGATGTAGGCCACGCCGC CGCCAGGGACG	
		P3: CGTCCCTGGCGGCGGCGTGGCCTACATCCGCTGCCTCAA GGCGCTGGACG	PCR amplification of C-terminal (1254bp to the end) of *groEL1*

		P4: CGGGATCCCTACATGCCCATACCGCCCATACCG	
	primer for the research of equatorial -C region of *groEL2*	p1: GCTCTAGAACACCTCCCGTCATTCCCCG	PCR amplification of upstream sequence (0.5kb)+the sequence of N-terminal (1-1254bp) of *groEL1*
		p2: TCTCCAGCGCGGGCAGCGCGCGGAGGTAGGCCACGCCGC CGCCAGGGACG	
		P3: CGTCCCTGGCGGCGGCGTGGCCTACCTCCGCGCGCTGCC CGCGCTGGAGA	PCR amplification of C-terminal (1254bp to the end) of *groEL2*
		P4: CGGGATCCTCAGTAGTCCATGTCGTCGCCGC	

* The underlined letters indicated the Restriction sites.
