# Supplementary material for: Mechanisms Involved in the Functional Divergence of Duplicated GroEL Chaperonins in Myxococcus xanthus DK1622
Source: PLoS Genet. 2013 Feb 21;9(2):e1003306. doi: 10.1371/journal.pgen.1003306 (PMC3578752; doi:10.1371/journal.pgen.1003306)
Supplement: Table S5 — The information of the identified substrates bound by GroEL1 and/or GroEL2. S5-1, Substrates of both GroEL1 and GroEL2. S5-2, Specific substrates of GroEL1. S5-3, Specific substrates of GroEL2. S5-4, Non-specific substrates of GroEL. (PDF) [file pgen.1003306.s010.pdf]

Table S5. List of primers for GGM region deletion and shorten

Oligonucleotide Primers	Oligonucleotide sequence (5'→3')	Reference
GGM deletion region of groEL1	P1: TCATCATCGCCGAGGACAT	PCR amplification of upstream homologous arm of groEL1 for deletion of 6 repeats of GGM sequence
	P2: GCTCTAGAGCCGGCGGGGAGGTCCTTCT	
	P3: GCTCTAGATAGTCCGCCCCTCACCCCG	PCR amplification of downstream homologous arm of groEL1 for deletion of 6 repeats of GGM sequence
	P4: CGTTGGTGGGTTGCTTCTC	
GGM swapping region of groEL1	P1: TCATCATCGCCGAGGACAT	PCR amplification of upstream homologous arm of groEL1 for swapping of 3 repeats of GGM sequence
	P2: GTAGTCCATGTCGTCGCCGCCGTACATGCCACCCATCCCGCCCA	
	P3: TACGGCGGCGACGACATGGACTACTAGTCCGCCCCTCACCCCG	PCR amplification of upstream homologous arm of groEL1 for swapping of 3 repeats of GGM sequence
	P4: CGTTGGTGGGTTGCTTCTC	
