# Supplementary material for: Mechanisms Involved in the Functional Divergence of Duplicated GroEL Chaperonins in Myxococcus xanthus DK1622
Source: PLoS Genet. 2013 Feb 21;9(2):e1003306. doi: 10.1371/journal.pgen.1003306 (PMC3578752; doi:10.1371/journal.pgen.1003306)
Supplement: Table S6 — List of primers for GGM region deletion and shorten. (PDF) [file pgen.1003306.s011.pdf]

Table S6. The information of the identified substrates bound by GroEL1 and/or GroEL2

Table S6-1. Substrates of both GroEL1 and GroEL2

Proteins Name	SwissProt ID	Mr(KDa)	PI	secondary feature
Putative lipoprotein	gi|108463713|gb|ABF88898.1|	11.4	6.03	
Hypothetical protein MXAN_2650	gi|108460953|gb|ABF86138.1|	11.9	11.4	
Hypothetical protein MXAN_4293	gi|108467769|gb|ABF92954.1|	16.4	6.29	
ATP synthase F1, delta subunit	gi|108467946|gb|ABF93131.1|	19.9	9.28	
Hypothetical protein MXAN_2526	gi|108463603|gb|ABF88788.1|	20.2	9.36	
Alkyl hydroperoxide reductase C	gi|108466489|gb|ABF91674.1|	20.7	5.97	αβ
Hypothetical protein MXAN_7407	gi|108467710|gb|ABF92895.1|	21.6	6.91	
Hypothetical protein MXAN_6187	gi|108467953|gb|ABF93138.1|	23.2	4.86	
Pilin	gi|108464954|gb|ABF90139.1|	23.4	5.25	
Vegetative protein	gi|108466760|gb|ABF91945.1|	23.6	8.54	
transporter, MotA/TolQ/ExbB proton channel family	gi|108467178|gb|ABF92363.1|	23.8	9.51	
Ribosomal protein L3	gi|108461186|gb|ABF86371.1|	24.5	10.03	
MotA/TolQ/ExbB proton channel family protein	gi|108465307|gb|ABF90492.1|	25.5	9.34	
MotA/TolQ/ExbB proton channel family protein	gi|108465592|gb|ABF90777.1|	25.9	5.59	
OmpA family protein	gi|108460955|gb|ABF86140.1|	26.3	4.76	αβ+1β
PspA/IM30 family protein(regulatory protein for phage-shock-protein operon)	gi|108464898|gb|ABF90083.1|	27.5	6.81	
Hypothetical protein MXAN_6976	gi|108462340|gb|ABF87525.1|	29.1	8.14	
3-deoxy-8-phosphooctulonate synthase	gi|108462440|gb|ABF87625.1|	30	7.05	αβ
Putative chemotaxis MotB protein	gi|108461220|gb|ABF86405.1|	31.8	9.09	αβ
Conserved hypothetical protein	gi|108462341|gb|ABF87526.1|	32	6.98	
ATP synthase F1, gamma subunit	gi|108461850|gb|ABF87035.1|	32.5	9.87	αβ+1α
Putative lipoprotein	gi|108462639|gb|ABF87824.1|	33.1	5.32	
Hypothetical protein MXAN_4860	gi|108463957|gb|ABF89142.1|	33.5	4.56	
ATPase, AAA family	gi|108464238|gb|ABF89423.1|	34.5	5.05	αβ
Putative isocitrate dehydrogenase, NAD-dependent	gi|108461038|gb|ABF86223.1|	35.8	6.35	αβ
DNA-directed RNA polymerase, alpha subunit	gi|108461974|gb|ABF87159.1|	37.6	5.42	αβ+1α+1β
Efflux transporter, RND family, MFP subunit	gi|108463443|gb|ABF88628.1|	38.7	9.01	
Putative lipoprotein	gi|108466690|gb|ABF91875.1|	39.1	5.69	αβ
Patatin-like phospholipase family protein	gi|108463726|gb|ABF88911.1|	39.3	8.24	αβ
GAF/GGDEF domain protein	gi|108461449|gb|ABF86634.1|	39.5	5.26	2αβ
4-hydroxyphenylpyruvate dioxygenase	gi|108464159|gb|ABF89344.1|	39.8	5.24	2αβ
Succinyl-CoA synthase, beta subunit	gi|108465422|gb|ABF90607.1|	41.2	6.08	3αβ
NAD dependent epimerase/dehydratase family	gi|108462464|gb|ABF87649.1|	41.9	6.35	αβ
Putative branched chain amino acid ABC transporter, periplasmic amino acid-binding protein	gi|108463874|gb|ABF89059.1|	42.1	6.66	αβ
Putative lipoprotein	gi|108464160|gb|ABF89345.1|	42.5	5.48	
Translation elongation factor Tu	gi|108467558|gb|ABF92743.1|	43.4	5.69	αβ+1β
Translation elongation factor Tu	gi|108462329|gb|ABF87514.1	43.4	5.69	αβ+1β
Aminotransferase, class I and II family protein	gi|108463704|gb|ABF88889.1|	44.6	6.28	2αβ

Acetyl-CoA carboxylase, biotin carboxylase	gi|108461326|gb|ABF86511.1|	51.1	6.19	3αβ
OB-fold nucleic acid binding domain protein	gi|108464424|gb|ABF89609.1|	51.1	8.51	
Putative lipoprotein	gi|108461727|gb|ABF86912.1|	53.1	4.43	
Hypothetical protein MXAN_4180	gi|108466301|gb|ABF91486.1|	53.3	5.87	
5'-nucleotidase family protein	gi|108460777|gb|ABF85962.1|	53.6	6.64	2αβ
Propionyl-CoA carboxylase, alpha subunit	gi|108466413|gb|ABF91598.1|	55.3	8.82	3αβ
Putative lipoprotein	gi|108463316|gb|ABF88501.1|	56.9	4.88	
Peptidylprolyl cis-trans isomerase	gi|108462303|gb|ABF87488.1|	57.7	5.62	αβ
DSBA-like thioredoxin domain protein	gi|108461115|gb|ABF86300.1|	59.5	8.66	αβ
ATP synthase F1, alpha subunit	gi|108465919|gb|ABF91104.1|	60.1	8.96	αβ+1α+1β
Hypothetical protein MXAN_5743	gi|108467878|gb|ABF93063.1|	64.8	7.04	
Fe-S protein assembly chaperone HscA	gi|108464420|gb|ABF89605.1|	65.7	5	αβ+1β
Putative lipoprotein	gi|108464035|gb|ABF89220.1|	66.3	4.25	
Putative long-chain-fatty-acid--CoA ligase	gi|108462896|gb|ABF88081.1|	66.7	7.74	αβ+1β
Succinate dehydrogenase, flavoprotein subunit	gi|108465170|gb|ABF90355.1|	69.2	7.26	2αβ+1α
Putative peptidase, S37 family	gi|108464098|gb|ABF89283.1|	71.2	4.27	
Chaperone protein HtpG	gi|108463905|gb|ABF89090.1|	73.3	5.66	αβ
PBS lyase HEAT-like repeat protein	gi|108467788|gb|ABF92973.1|	73.7	5.64	
Prolyl endopeptidase precursor Pep	gi|108464609|gb|ABF89794.1|	76.8	6.55	αβ+1α
Peptidase, M13 (neprilysin) family	gi|108462397|gb|ABF87582.1|	78.5	6.57	2αβ+1α
Matrix-associated zinc metalloprotease FibA	gi|108465800|gb|ABF90985.1|	79.8	5.2	αβ+1α
ATP-dependent protease La	gi|108467702|gb|ABF92887.1|	90.4	5.85	αβ+1α
Preprotein translocase, SecA subunit	gi|108463299|gb|ABF88484.1|	106.3	5.94	3αβ+1α
Putative lipoprotein	gi|108465910|gb|ABF91095.1	107.3	4.83	
Translation initiation factor IF-2	gi|108463647|gb|ABF88832.1|	111.9	9.22	2αβ+1β
TonB-dependent receptor	gi|108467528|gb|ABF92713.1|	122.4	5.84	
B12 binding domain/ArgK/methylmalonyl-CoA mutase family protein	gi|108464917|gb|ABF90102.1|	128.5	6.03	3αβ
DNA-directed RNA polymerase, beta' subunit	gi|108463590|gb|ABF88775.1|	156.3	7.54	1β
DNA-directed RNA polymerase, beta subunit	gi|108465184|gb|ABF90369.1|	157.5	5.56	αβ+3β
Conserved hypothetical protein	gi|108466233|gb|ABF91418.1|	164.5	5.51	

Table S6-2. Specific substrates of GroEL1

Proteins Name	SwissProt ID	Mr（KDa）	pI	Secondary feature
Hypothetical protein MXAN_3135	gi|108461349|gb|ABF86534.1|	13	12.04	
Transport energizing protein, ExbD/TolR family	gi|108466289|gb|ABF91474.1|	15.4	4.45	
Transport energizing protein, ExbD/TolR family	gi|108464717|gb|ABF89902.1|	15.4	4.54	
Hypothetical protein MXAN_6084	gi|108464051|gb|ABF89236.1|	16.6	5.51	
Hypothetical protein MXAN_0662	gi|108464316|gb|ABF89501.1|	18.7	8.78	
Hypothetical protein MXAN_5567	gi|108461837|gb|ABF87022.1|	18.8	4.79	
ATP synthase F0, B subunit	gi|108464094|gb|ABF89279.1|	19.4	9.18	
Vegetative protein	gi|108465050|gb|ABF90235.1|	19.4	10.38	
Acetyl-CoA carboxylase, biotin carboxyl carrier protein	gi|108467252|gb|ABF92437.1|	21	5.98	1β
Hypothetical protein MXAN_5512	gi|108467881|gb|ABF93066.1|	21.5	5.51	
Cytochrome c family protein	gi|108464492|gb|ABF89677.1|	22	6.43	1α
Putative lipoprotein	gi|108461894|gb|ABF87079.1|	22	5.14	
Putative lipoprotein	gi|108465829|gb|ABF91014.1|	23.1	4.34	
Ribosomal 5S rRNA E-loop binding protein Ctc/L25/TL5	gi|108467517|gb|ABF92702.1|	23.3	9.64	2β
Conserved hypothetical protein	gi|108467759|gb|ABF92944.1|	23.4	5.3	
Conserved hypothetical protein	gi|108463349|gb|ABF88534.1|	23.6	10.88	
Putative cytochrome c	gi|108461323|gb|ABF86508.1|	24.6	6.48	
Putative lipoprotein MlpA	gi|108465162|gb|ABF90347.1|	24.6	4.41	
Ribosomal protein S3	gi|108465909|gb|ABF91094.1|	24.8	10.06	2αβ
Hypothetical protein MXAN_6660	gi|108467412|gb|ABF92597.1|	25.3	4.51	
Hypothetical protein MXAN_5716	gi|108461439|gb|ABF86624.1|	25.8	8.82	
Glycosyl transferase, group 2 family protein	gi|108466337|gb|ABF91522.1|	26	6.44	αβ
Oxidoreductase, short chain dehydrogenase/reductase family	gi|108467801|gb|ABF92986.1|	26.3	8.71	αβ
Circadian phase modifier CpmA-like protein	gi|108465659|gb|ABF90844.1|	26.3	9.39	
Putative TonB protein	gi|108466663|gb|ABF91848.1|	26.5	5.88	
Peptidyl-prolyl cis-trans isomerase, FKBP-type	gi|108466899|gb|ABF92084.1|	27.3	6.77	αβ
Hypothetical protein MXAN_5530	gi|108464256|gb|ABF89441.1|	27.5	9.86	
Conserved hypothetical protein TIGR00266	gi|108462992|gb|ABF88177.1|	27.9	5.11	
D-lysine 5,6-aminomutase, beta subunit	gi|108460697|gb|ABF85882.1|	28.7	8.42	2αβ
Hypothetical protein MXAN_0572	gi|108467148|gb|ABF92333.1|	29.2	4.69	
Putative Flp pilus assembly protein CpaB	gi|108462905|gb|ABF88090.1|	29.5	6.23	
Hypothetical protein MXAN_5235	gi|108465621|gb|ABF90806.1|	31.5	9.99	
Hypothetical protein MXAN_3556	gi|108462612|gb|ABF87797.1|	32.5	5.43	
Kinase, pfkB family	gi|108467360|gb|ABF92545.1|	33.9	6.12	αβ
Putative cysteine synthase La	gi|108460810|gb|ABF85995.1|	34.1	6.91	αβ
Hypothetical protein MXAN_5056	gi|108465950|gb|ABF91135.1|	34.1	9.75	
NAD-dependent epimerase/dehydratase family protein	gi|108461556|gb|ABF86741.1|	34.8	6.25	αβ
NAD dependent epimerase/dehydratase family protein	gi|108464298|gb|ABF89483.1|	35.7	9.54	αβ
Conserved hypothetical protein	gi|108466780|gb|ABF91965.1|	36.6	6.57	
GDP-mannose 4,6-dehydratase	gi|108464244|gb|ABF89429.1|	36.8	6.57	2αβ

Hypothetical protein MXAN_7193	gi|108462848|gb|ABF88033.1|	37.2	10.51	
Cytochrome c oxidase, subunit II	gi|108465540|gb|ABF90725.1|	39.4	5.93	1β
Efflux transporter, HAE1 family, MFP subunit	gi|108465925|gb|ABF91110.1|	39.7	9.32	1β
efflux transporter, RND family, MFP subunit	gi|108462604|gb|ABF87789.1|	41.2	9.09	
Putative outer membrane protein P1	gi|108465243|gb|ABF90428.1|	42.1	5.83	1β
Hypothetical protein MXAN_6152	gi|108467500|gb|ABF92685.1|	42.5	10.21	
Conserved hypothetical protein	gi|108460963|gb|ABF86148.1|	43.3	8.8	
Putative serine/threonine protein kinase	gi|108460939|gb|ABF86124.1|	43.6	9.63	1α
Frizzy aggregation protein FrzCD	gi|108461945|gb|ABF87130.1|	43.6	5.17	1α
Transcription termination factor Rho	gi|108464434|gb|ABF89619.1|	47	6.54	αβ+1β
Conserved hypothetical protein	gi|108467288|gb|ABF92473.1|	47.5	5.1	
Peptidase, S1C (protease Do) subfamily	gi|108460906|gb|ABF86091.1|	47.7	9.11	2β
Putative glutamate-cysteine ligase	gi|108462861|gb|ABF88046.1|	49.1	6	
Pyruvate dehydrogenase complex , E2 component, dihydrolipoamide acetyltransferase	gi|108463562|gb|ABF88747.1|	55.1	9.23	αβ+1β
Putative lipoprotein	gi|108464216|gb|ABF89401.1|	57.6	4.99	
Endonuclease/exonuclease/phosphatase family protein	gi|108465412|gb|ABF90597.1|	61	4.75	
Putative ABC transporter, permease/ATP-binding protein	gi|108461711|gb|ABF86896.1|	63.2	9.17	αβ+1α
Hypothetical protein MXAN_1792	gi|108467622|gb|ABF92807.1|	63.8	5.57	
Putative lipoprotein	gi|108465712|gb|ABF90897.1|	64.3	8.35	
Chaperone protein DnaK	gi|108464899|gb|ABF90084.1|	65.3	5.15	3αβ+1α
V-type H(+)-translocating pyrophosphatase	gi|108463776|gb|ABF88961.1|	69.7	5.63	
FHA domain protein	gi|108463173|gb|ABF88358.1|	77.5	9.81	
Conserved domain protein	gi|108463858|gb|ABF89043.1|	79.1	8.14	
Putative alkaline phosphatase	gi|108461003|gb|ABF86188.1|	81.4	5.06	1α
ATP-dependent helicase, DEAD/DEAH-box family	gi|108467215|gb|ABF92400.1|	89	6.28	αβ
Sensor histidine kinase/response regulator CheA4	gi|108465504|gb|ABF90689.1|	89.4	5.88	2αβ+1β
ATP-dependent protease	gi|108467808|gb|ABF92993.1|	91.8	9.09	αβ+α
General secretion pathway protein D	gi|108461310|gb|ABF86495.1|	91.8	5.58	
Sensor histidine kinase/response regulator	gi|108467866|gb|ABF93051.1|	92	5.93	2αβ
ATP-dependent helicase HrpB	gi|108465444|gb|ABF90629.1|	93.4	8.79	αβ
Type IV pilus secretin PilQ	gi|108463309|gb|ABF88494.1|	95.7	9.49	
DNA polymerase	gi|108463657|gb|ABF88842.1|	99.6	6.16	3αβ+2α
TonB-dependent receptor	gi|108461839|gb|ABF87024.1|	103.4	5.98	
RND transporter, hydrophobe/amphiphile efflux-1 (HAE1) family	gi|108461042|gb|ABF86227.1|	111.5	5.28	2αβ+2α
Putative molybdopterin oxidoreductase, iron-sulfur binding subunit	gi|108466940|gb|ABF92125.1|	116.3	6.18	αβ
Polyketide synthase type	gi|108462423|gb|ABF87608.1|	150.2	6.01	2αβ
Putative long-chain-fatty-acid CoA ligase	gi|108462818|gb|ABF88003.1|	162.3	6.6	1β
Non-ribosomal peptide synthase MxaA	gi|108465274|gb|ABF90459.1|	163	6.07	2αβ+1α+1β
Rhs family protein	gi|108466825|gb|ABF92010.1|	194	5.37	1β
Polyketide synthase type I	gi|108464516|gb|ABF89701.1|	199.3	6	2αβ
Sensor histidine kinase/response regulator	gi|108460885|gb|ABF86070.1|	212.7	5.49	2αβ
Non-ribosomal peptide synthase/polyketide synthase	gi|108467361|gb|ABF92546.1|	324.1	5.86	3αβ
Adventurous gliding motility protein AgmK	gi|108461297|gb|ABF86482.1|	421.5	5.18	1α

Table S6-3. Specific substrates of GroEL2

Proteins Name	SwissProt ID	Mr（KDa）	pI	Secondary feature
Hypothetical protein MXAN_1734	gi|108464654|gb|ABF89839.1|	5.9	11.6	
Hypothetical protein MXAN_2720	gi|108466490|gb|ABF91675.1|	10.5	8.18	
Hypothetical protein MXAN_4368	gi|108466097|gb|ABF91282.1|	14.5	10.28	
Hypothetical protein MXAN_5286	gi|108460772|gb|ABF85957.1|	18.1	10.55	
gliding motility protein MglA	gi|108462799|gb|ABF87984.1|	22	8.94	αβ
DnaJ domain protein	gi|108466045|gb|ABF91230.1|	24.8	8.47	1α
Vegetative protein	gi|108463008|gb|ABF88193.1|	27.7	11.43	
2-oxo acid dehydrogenase acyltransferase catalytic domain protein	gi|108463708|gb|ABF88893.1|	32	9.21	αβ
Pyruvate dehydrogenase complex, E1 component, pyruvate dehydrogenase, beta subunit	gi|108462490|gb|ABF87675.1|	35.5	5.1	2αβ
Rod shape-determining protein MreB	gi|108465738|gb|ABF90923.1|	36.5	5.26	3αβ
Acyl-CoA dehydrogenase	gi|108460671|gb|ABF85856.1|	41	5.86	2α+1β
Acetyl-CoA acetyltransferase family protein	gi|108466785|gb|ABF91970.1|	41.6	5.95	αβ
Type IV pilus biogenesis protein PilM	gi|108463407|gb|ABF88592.1|	42.2	4.82	αβ
Conserved domain protein	gi|108466054|gb|ABF91239.1|	42.5	6.68	
Putative aspartate aminotransferase	gi|108464559|gb|ABF89744.1	42.9	6.78	2αβ
Pyruvate dehydrogenase complex, E1 component, pyruvate dehydrogenase, alpha subunit	gi|108465752|gb|ABF90937.1	43.7	5.56	αβ
Isocitrate dehydrogenase, NADP-dependent	gi|108463607|gb|ABF88792.1|	47.3	6.64	αβ
Peptidase, M16 (pitrilysin) family	gi|108464326|gb|ABF89511.1|	51.1	8.4	αβ
ATP synthase F1, beta subunit	gi|108463470|gb|ABF88655.1|	51.8	5.02	αβ+1α+1β
Protein transporter, outer bacterial membrane secretin (secretin) family	gi|108466805|gb|ABF91990.1|	51.8	9.12	
Hypothetical protein MXAN_5216	gi|108462406|gb|ABF87591.1|	52.4	10.19	αβ
Sigma-54 dependent DNA-binding response regulator Nla19	gi|108467699|gb|ABF92884.1|	53.2	6.56	2αβ+1α
Glycerol kinase	gi|108466269|gb|ABF91454.1|	53.8	9.19	αβ
Glutamate--ammonia ligase	gi|108463107|gb|ABF88292.1|	54.4	6.37	2αβ
Hypothetical protein MXAN_2659	gi|108466474|gb|ABF91659.1|	55.6	5.49	
Peptidase, M16 (pitrilysin) family	gi|108466919|gb|ABF92104.1|	58.7	5.93	αβ
Poly(A) polymerase	gi|108464894|gb|ABF90079.1|	60	4.82	αβ
Conserved hypothetical protein	gi|108466266|gb|ABF91451.1|	62	8.15	αβ
Type IV-A pilus assembly ATPase PilB	gi|108464603|gb|ABF89788.1|	62.5	5.22	2αβ
Phosphoenolpyruvate carboxykinase (GTP)	gi|108461634|gb|ABF86819.1|	66.1	6.26	2αβ+1β
HAMP domain/GAF domain/GGDEF domain protein	gi|108467379|gb|ABF92564.1|	70	6.07	2αβ
Hypothetical protein MXAN_6253	gi|108465637|gb|ABF90822.1|	72	9.03	
Putative acyl-CoA dehydrogenase	gi|108462001|gb|ABF87186.1|	72.7	6.7	1α+1β
Peptidase, M3 (thimet oligopeptidase) family	gi|108461854|gb|ABF87039.1	72.9	6.12	αβ+1α
Tetratricopeptide repeat protein	gi|108461871|gb|ABF87056.1|	76	8.82	
Methionyl-tRNA synthetase	gi|108466716|gb|ABF91901.1	77.3	5.98	αβ+1α+3β
Excinuclease ABC, B subunit	gi|108463428|gb|ABF88613.1|	79.2	5.58	αβ
3-hydroxyacyl-CoA dehydrogenase	gi|108464934|gb|ABF90119.1|	86.4	6.85	2αβ
Penicillin acylase family protein	gi|108464049|gb|ABF89234.1|	88.9	5.14	αβ+2α
Hypothetical protein MXAN_4986	gi|108466839|gb|ABF92024.1|	108.7	6.02	

DNA polymerase III, alpha subunit	gi|108465847|gb|ABF91032.1|	111.3	7.22	
Carbamoyl-phosphate synthase, large subunit	gi|108467618|gb|ABF92803.1|	118.8	5.48	3αβ+1α
Putative non-ribosomal peptide synthetase	gi|108461374|gb|ABF86559.1|	130.2	5.65	αβ+1α+1β
Conserved domain protein	gi|108466076|gb|ABF91261.1|	132.2	8.49	αβ
Polyketide synthase type	gi|108463875|gb|ABF89060.1|	231.9	5.76	3αβ
Non-ribosomal peptide synthetase	gi|108467458|gb|ABF92643.1|	495.2	5.66	3αβ+1α+1β

Table S6-4. Non-specific substrates of GroEL

Proteins Name	SwissProt ID
translation elongation factor Tu	gi|108467558|gb|ABF92743.1|
conserved hypothetical protein	gi|108464213|gb|ABF89398.1|
ribosomal protein S1	gi|108467641|gb|ABF92826.1|
ribosomal protein S2	gi|108462257|gb|ABF87442.1|
ribosomal protein L5	gi|108461461|gb|ABF86646.1|
ribosomal protein L2	gi|108463768|gb|ABF88953.1|
ribosomal protein L4	gi|108465431|gb|ABF90616.1|
ribosomal protein L10	gi|108466139|gb|ABF91324.1|
ribosomal protein S12	gi|108462449|gb|ABF87634.1|
ribosomal protein S4	gi|108462544|gb|ABF87729.1|
hypothetical protein MXAN_7407	gi|108467710|gb|ABF92895.1|
alkyl hydroperoxide reductase C	gi|108466489|gb|ABF91674.1|
ATP synthase F1, beta subunit	gi|108463470|gb|ABF88655.1|
ribosomal protein S4	gi|108462544|gb|ABF87729.1|
conserved hypothetical protein	gi|108464297|gb|ABF89482.1|
ribosomal protein L3	gi|108461186|gb|ABF86371.1|
ribosomal protein S8	gi|108463341|gb|ABF88526.1|
ribosomal protein L19	gi|108466655|gb|ABF91840.1|
ribosomal protein L18	gi|108463024|gb|ABF88209.1|
ribosomal protein L9	gi|108464695|gb|ABF89880.1|
ribosomal protein L15	gi|108462535|gb|ABF87720.1|
ribosomal protein S7	gi|108467649|gb|ABF92834.1|
